# Efficient Isolation and Enrichment of Mesenchymal Stem Cells from Human Embryonic Stem Cells by Utilizing the Interaction between Integrin *α*5*β*1 and Fibronectin

**DOI:** 10.1002/advs.202001365

**Published:** 2020-07-19

**Authors:** Byung‐Hyun Cha, Jin‐Su Kim, Alvin Bello, Geun‐Hui Lee, Do‐Hyun Kim, Byoung Ju Kim, Yoshie Arai, Bogyu Choi, Hansoo Park, Soo‐Hong Lee

**Affiliations:** ^1^ Division of Cardio‐Thoracic Surgery Department of Surgery College of Medicine University of Arizona Tucson AZ 85724 USA; ^2^ CellenGene R&D Center Open Innovation Building Seoul 02455 Republic of Korea; ^3^ Department of Biomedical Science CHA University CHA Biocomplex Seongnam‐si Gyeonggi‐do 13488 Republic of Korea; ^4^ Department of Integrative Engineering Chung‐Ang University Seoul 06974 Republic of Korea; ^5^ Department of Medical Biotechnology Dongguk University 32 Dongguk‐ro, Ilsandong‐gu Goyang Gyeonggi 10326 Republic of Korea

**Keywords:** fibronectin, human embryonic stem cells, integrin, mesenchymal stem cells, stem cell therapeutics

## Abstract

Human pluripotent stem cells (hPSCs) are a potent source of clinically relevant mesenchymal stem cells (MSCs) that confer functional and structural benefits in cell therapy and tissue regeneration. Obtaining sufficient numbers of MSCs in a short period of time and enhancing the differentiation potential of MSCs can be offered the potential to improve the regenerative activity of MSCs therapy. In addition, the underlying processes in the isolation and derivation of MSCs from hPSCs are still poorly understood and controlled. To overcome these clinical needs, an efficient and simplified technique on the isolation of MSCs from spontaneously differentiated human embryonic stem cells (hESCs) via integrin *α*5*β*1 (fibronectin (FN) receptor)‐to‐FN interactions (hESC‐FN‐MSCs) is successfully developed. It is demonstrated that hESC‐FN‐MSCs exhibit a typical MSC surface phenotype, cellular morphology, with the whole transcriptome similar to conventional adult MSCs; but show higher proliferative capacity, more efficient trilineage differentiation, enhanced cytokine secretion, and attenuated cellular senescence. In addition, the therapeutic potential and regenerative capacity of the isolated hESC‐FN‐MSCs are confirmed by in vitro and in vivo multilineage differentiation. This novel method will be useful in the generation of abundant amounts of clinically relevant MSCs for stem cell therapeutics and regenerative medicine.

## Graphical abstract

Efficient isolation of mesenchymal stem cells (MSCs) out of a heterogeneous mixture of differentiating cells from human embryonic stem cells (hESCs) is technically challenging. A novel method is developed, in which integrin α5β1‐mediated interactions with fibronectin are utilized to isolate homogeneous MSCs from spontaneously differentiating hESCs. This novel method will be useful to secure clinically relevant MSC numbers for stem cell therapeutics.

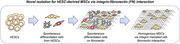

## 1 Introduction

To date, various mesenchymal stem cells (MSCs) have been widely utilized in clinical trials, even though MSCs have limited proliferation, donor variations, and scale‐up potential for cell therapeutics and regenerative medicine applications. For the last few years, several methods have been developed for the derivation and isolation of human embryonic stem cells (hESCs)^[^ ^1^ ^]^ and induced pluripotent stem cell (hiPSC)‐derived MSCs^[^ ^2^, ^3^ ^]^ as alternative potential cell sources to overcome the disadvantages and limitations of MSCs derived from various tissues.^[^ ^4^ ^]^ However, these methodologies are ultimately inadequate for clinical application due to their low isolation efficiency, low differentiation ability, heterogeneous cell population, and high cost.^[^ ^5^ ^]^

Although the process of mesoderm formation during early development is well understood, differentiation and isolation techniques for MSCs and mesodermal progenitor‐like cells from pluripotent stem cells (PSCs), such as hESCs and hiPSCs, remain poorly developed. Representatively, selected small‐molecules,^[^ ^6^ ^]^ spontaneously differentiating embryoid bodies (EBs),^[^ ^2^, ^7^ ^]^ cytokine/growth factor combinations,^[^ ^8^, ^9^ ^]^ coculturing systems,^[^ ^10^ ^]^ and fluorescence‐activated cell sorting (FACS) after MSCs derivation/differentiation^[^ ^2^, ^5^ ^]^ have been reported. In addition, simplified techniques using physical cues, such as biomimetic fibrillar collagen,^[^ ^5^ ^]^ gelatin,^[^ ^2^ ^]^ and Matrigel,^[^ ^2^, ^11^ ^]^ have been utilized to derive MSCs from PSCs. However, unfortunately, the efficient and simplified methods are yet to be fully established to overcome the limitations of previous methods.

To expand upon the concept of simplified techniques for MSCs isolation via physical interactions between substrates and MSCs, the MSC‐integrin dynamics and substrate assembly must be understood. Integrins are substrate‐responsive signaling receptors that bind to extracellular matrix (ECM) ligands, cell‐surface ligands, and soluble ligands.^[^ ^12^ ^]^ They are implicated in the control of various cellular processes including cell attachment, spreading, motility, proliferation, differentiation, and apoptosis.^[^ ^13^ ^]^ According to the literature, human MSCs highly express *α*1, *α*2, *α*3, *α*5, *α*V, *β*1, *β*3, and *β*5 integrin subunits^[^ ^14^ ^]^ and bind to fibronectin (FN) via *α*5*β*1 (FN binding receptor), vitronectin via *α*v*β*3, laminin via *α*6*β*1, and collagens I, III, and IV via *α*1*β*1 and *α*2*β*1 integrins.^[^ ^15^ ^]^ We also confirmed the dominant expression of *α*5*β*1 integrin in adult MSCs in the Supporting Information. FN contains an arginine‐glycine‐aspartic acid (RGD) sequence that improves cell adhesion to FN‐coated surfaces via integrin *α*5*β*1.^[^ ^16^ ^]^ In addition to the RGD motif, FN contains other integrin‐mediated cell binding motifs including Lys‐Gln‐Ala‐Gly‐Asp‐Val (KQAGDV), Arg‐Glu‐Asp‐Val (REDV), and Pro‐His‐Ser‐Arg‐Am (PHSRN).^[^ ^17^ ^]^

During gastrulation and vertebrate development, FN, one of the ECM proteins, plays a crucial role in the development of mesoderm and mesoderm‐derived structures.^[^ ^18^ ^]^ George et al. reported that FN‐deficient mice died on embryonic days 8–8.5 due to several mesodermal defects, including a disorganized notochord, absence of a heart and somites, and abnormal vasculogenesis.^[^ ^19^ ^]^ Other studies have also demonstrated that inhibition of integrin *α*5*β*1–FN interactions resulted in abnormalities in early gastrulation and mesoderm formation.^[^ ^19^, ^20^ ^]^ As experimental evidence, Pimton et al. found that FN can mediate mouse ESC differentiation toward a mesodermal lineage by upregulating the expression of integrin *α*5*β*1.^[^ ^21^ ^]^ These findings are interesting in this context because integrin *α*5*β*1–FN interactions convey essential signals from the beginning of gastrulation to mesoderm development. Based on the expression of integrin *α*5*β*1 in MSCs, we hypothesized that integrin *α*5*β*1‐mediated interactions with FN can be harnessed as an efficient method to isolate homogeneous/uniformed MSCs from spontaneously differentiating hESCs (SD‐hESCs).

In this study, we developed the efficient and simplified methods for the creation of homogeneous/uniformed MSCs from SD‐hESCs via integrin *α*5*β*1‐mediated interactions with FN. Compared to human adipose‐derived mesenchymal stem cells (ASCs) and bone marrow‐derived mesenchymal stem cells (BMMSCs), FN‐mediated hESC‐derived MSCs (hESC‐FN‐MSCs) show typical MSC characteristics, with improved proliferation and cytokine secretion, as well as the attenuation of senescence status. In vitro and in vivo studies showed that hESC‐FN‐MSCs are capable of multipotent differentiation and tissue regeneration. Our methods also indicate that hESC‐MSCs selected by FN substrates are promising MSC sources for clinical trials and regenerative medicine applications.

## 2 Results and Discussion

**Figure 1:**
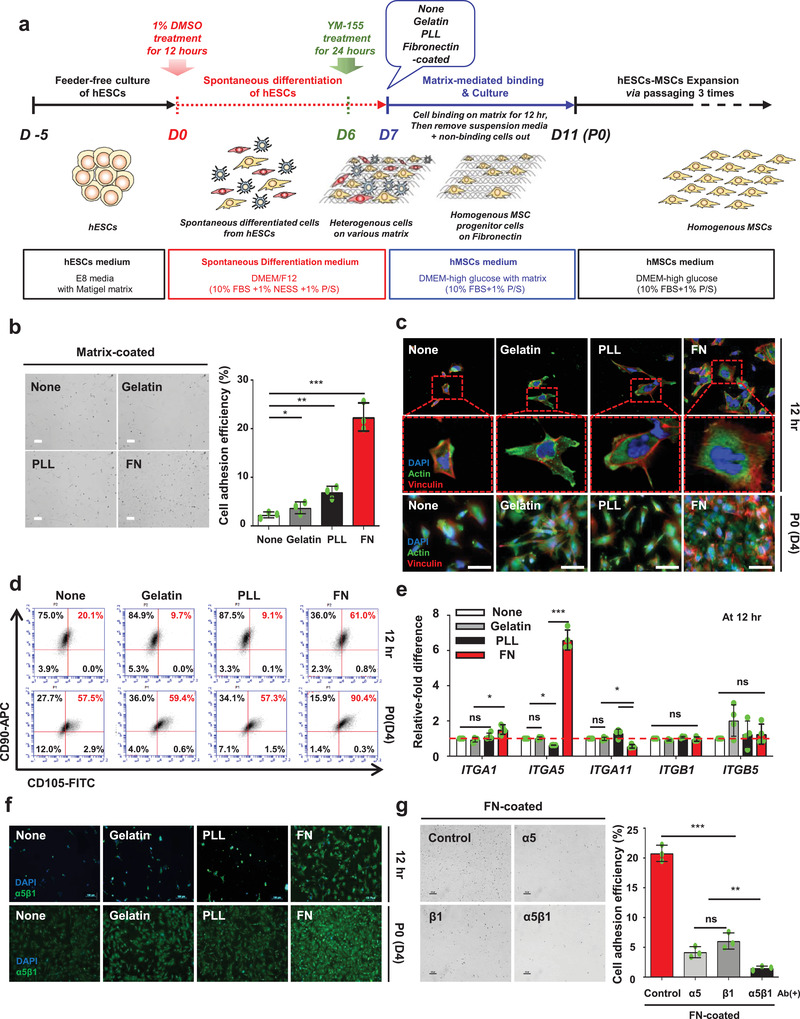
Human ESC‐derived MSC‐like cells (CD90^+^CD105^+^ cells) can be isolated via integrin *α*5*β*1–FN‐mediated interactions. a) Schematic depicting the overall procedure for hESC‐derived MSCs via cell–FN‐mediated interactions. b) FN‐coated group shows relatively higher level of adhesion efficiency for single spontaneously differentiated hESCs compared to the other groups. Scale bar: 100 µm. *n* = 3, mean ± s.d., ^*^ *P* < 0.05, ^**^ *P* < 0.01, and ^***^ *P* < 0.001 (one‐way analysis of variance (ANOVA)). c) After 7 days of spontaneous differentiation of hESCs, followed by matrix‐mediated binding for 12 h, actin and vinculin staining at P0 (day 4) showed that the highest cell binding and spreading occurred in the FN‐coated group but not in the other groups. Scale bar: 100 µm. d) FACS analysis of the mesenchymal stem cell markers CD90 and CD105 at 12 h and P0 (day 4) after matrix‐mediated binding, FN‐coated group shows significantly higher level of positive CD90 and CD105 cells compared to the other groups. e) At 12 h after matrix‐mediated binding, the expression of *ITGA5* is significantly increased in cells on FN compared to the cells on other matrices, whereas *ITGA1*, *ITGA11*, *ITGB1*, and *ITGB5* expression in all substrates showed no difference. None (control) is normalized to 1. *n* = 3, mean ± s.d., ns, not significant, ^*^ *P* < 0.05, and ^***^ *P* < 0.001 (two‐way ANOVA). f) At 12 h and P0 (day 4) after matrix‐mediated binding, FN‐coated group shows relatively higher level of integrin *α*5*β*1 expression with more cell adhesion compared to the other groups. Scale bar: 100 µm. g) The cells were subjected to integrins *α*5, *β*1, and *α*5*β*1 inhibition using antibodies prior to FN‐mediated binding. All integrins *α*5, *β*1, and *α*5*β*1 Ab‐inhibited cells in the FN‐coated group exhibited decreased cell adhesion efficiency. Interestingly, cells subjected to integrin *α*5*β*1 inhibition showed the lowest cell adhesion compared to those with integrins *α*5 and *β*1 inhibition. Scale bar: 100 µm. *n* = 3, mean ± s.d., ns, not significant, ^**^ *P* < 0.01, and ^***^ *P* < 0.001 (one‐way ANOVA).

The utilization of ECMs to develop efficient methods to isolate MSCs or mesodermal progenitor‐like cells from hESCs and hiPSCs remains largely unexplored.^[^ ^2^, ^5^, ^11^ ^]^ Fibronectin, FN is a large ECM glycoprotein that triggers biochemical and mechanical signaling via integrin binding. Another hypothesis for the efficacy of this method is ECM remodeling in the earliest stages of embryonic development.^[^ ^22^ ^]^ The FN substrate is essential and plays a crucial role in mesoderm derivatives through mesodermal cell condensation.^[^ ^18^ ^]^ Therefore, we hypothesized that the FN substrate can be utilized to isolate mesodermal progenitor‐like cells or MSCs from PSCs such as hESCs or hiPSCs. Accordingly, as shown in **Figure** **1**a, we designed a novel isolation protocol, combined with spontaneously differentiating ESCs (SD‐ESCs) and a ECM‐mediated binding selection step, which differs from cytokine cocktail‐based differentiation protocols.^[^ ^8^ ^]^ To determine the optimal period for spontaneous differentiation from hESCs, we examined the changes in the expression of three germ layer lineage (endoderm, mesoderm, and ectoderm)‐related genes during the progression of spontaneous differentiation from hESCs (Figure S1a, Supporting Information). We found that mesoderm lineage‐related markers such as *Brachy* (2.49 ± 0.49 ng µL^−1^ of total RNA) and *Slug/Snail* (3.39 ± 0.01 ng µL^−1^) were highly expressed on day 7 compared to their expression on day 5 and day 9 (*Brachy* and *Slug/Snail* on day 5: 1.11 ± 0.31 and 1.00 ± 0.01 ng µL^−1^; on day 9: 0.90 ± 0.17 and 0.79 ± 0.01 ng µL^−1^). Exceptionally, one of the endoderm‐lineage related markers, *Krt19*, showed a similar trend as *Brachy* and *Slug/Snail*, whereas the other endoderm and ectoderm‐related markers showed a modest trend of lower expression levels compared to *Brachy* and *Slug/Snail* on day 7. Therefore, hESCs were cultured in a feeder‐free system with Matrigel for spontaneous differentiation over 7 days. Before culturing for spontaneous differentiation, we observed a typical colony of undifferentiated hESCs (Figure S1b‐b′, Supporting Information). After 7 days of spontaneous differentiation, we observed heterogeneous morphology and loss of the morphology of a typical undifferentiated hESC colony (Figure S1b‐b″, Supporting Information). According to flow cytometry analysis (Figure S1b‐b‴, Supporting Information), only 18.10% of the SD‐hESCs were double‐positive for CD90^+^ and CD105^+^,^[^ ^23^, ^24^ ^]^ so‐called specific MSCs surface markers. Spontaneous differentiation of hESCs can lead to a heterogeneous population of lineage‐specific differentiated cells including undifferentiated cells. In detail, hESCs were cultured with a feeder‐free culture system for 5 days. Spontaneous differentiation of hESCs for 7 days was performed with 1% dimethyl sulfoxide (DMSO) treatment (to enhance mesodermal lineage differentiation)^[^ ^25^ ^]^ for 12 h in the early stage of differentiation and YM‐155 treatment (to eliminate undifferentiated hESCs)^[^ ^26^ ^]^ for 1 day before the end of differentiation (Figure S2, Supporting Information). In the next step, the single SD‐ESCs dissociated with enzymatic methods were subcultured on different matrixes, specifically none‐coated, gelatin‐coated (conventionally used ECM), poly‐l‐lysine (PLL)‐coated (no integrin‐mediated binding caused by electrostatic interactions),^[^ ^27^ ^]^ and FN‐coated (mainly integrin *α*5*β*1‐mediated binding)^[^ ^28^ ^]^ tissue culture plates for 12 h. After selection with 12 h cell–matrix interaction, nonadherent cells were washed out. For in vitro expansion, the selected cells were serially passaged up to passage 3. At 12 h after matrix‐mediated binding, the FN‐mediated group showed a significantly higher level of cell adhesion efficiency (22.4 ± 2.88%) compared to the other groups (Figure 1b). As shown in Figure 1c, the focal adhesion formation of cells was observed after 12 h and at passage 0 (P0, 4 days after matrix‐mediated binding) after seeding on none‐coated, gelatin‐coated, PLL‐coated, and FN‐coated dishes, respectively. Among the cells cultured on FN for 12 h binding, the cell body was widely spread, exhibiting a fibroblastic morphology, and the actin filaments were well organized over the entire cell body. On the other hand, the cells cultured on none‐coated, gelatin‐coated, and PLL‐coated plates had a round and distorted body shape and the actin filaments were not well organized. Interestingly, the cells cultured on FN showed high adhesion efficiency with well‐organized actin filaments and vinculin. Next, we first set out to investigate whether integrin *α*5*β*1‐mediated interactions would be helpful to enrich CD90^+^CD105^+^ cells, MSC‐like cells, through comparative studies with different matrices. After 7 days of spontaneous differentiation, the proportion of CD90^+^CD105^+^ cells obtained from the hESCs was 18.1% (Figure S1b, Supporting Information). Spontaneous differentiation gave rise to a heterogeneous population containing mesoderm, endoderm, ectoderm, and even undifferentiated hESCs. When these single SD‐hESCs were exposed to the four substrates tested for 12 h, the FN‐incubated group contained the highest level of CD90^+^CD105^+^ double‐positive cells (61.0%) (Figure 1d; none‐coated: 20.1%; gelatin: 9.7%; PLL: 9.1%). After 4 days of incubation, the number of CD90^+^CD105^+^ cells on all of the substrates increased compared to the numbers at 12 h. These results demonstrate that the proportion of CD90^+^CD105^+^ cells produced on each substrate gradually increased as the incubation progressed. The FN substrate produced the highest number of CD90^+^CD105^+^ cells (90.4% on day 4). To determine the optimal cell‐binding duration for the cell‐to‐matrix‐mediated interactions, we investigated both cell adhesion efficiency and CD90^+^CD105^+^ cells in a time‐dependent manner during a 24 h period (Figure S3, Supporting Information). FN‐mediated CD90^+^CD105^+^ cell production was markedly and effectively higher at 12 h compared to production on the other matrices and at other time points. Furthermore, to confirm the correlation between actual integrin profiles of adult MSCs, such as ASCs and BMMSCs, and FN‐mediated CD90^+^CD105^+^ cell production from our novel method, we explored the integrin profile of adult MSCs by quantitative polymerase chain reaction (qPCR) (Figure S4a, Supporting Information). This result indicates that *ITGA1*, *ITGA5*, *ITGA11*, *ITGB1*, and *ITGB5* were dominantly expressed in both MSCs. Interestingly, these five representative MSC integrins were not highly expressed in FN‐mediated cells compared with other substrate‐mediated cells (Figure 1e). However, only FN‐mediated cells distinctly increased *ITGA5* expression. As shown in Figure S4b in the Supporting Information, integrin *α*5 and integrin *β*1 proteins were dominantly expressed in both ASCs and BMMSCs (ASCs: integrin *α*5: 95.8%, integrin *β*1: 99.6%; BMMSCs: integrin *α*5: 98.3%, integrin *β*1: 99.8%), implying that the dimer subunits of integrin *α*5 and integrin *β*1, as FN receptors, are predominantly expressed in both ASCs and BMMSCs. Additionally, at both 12 h and P0, FN‐mediated cells had the highest number of attached cells as well as the highest expression of integrin *α*5*β*1 (Figure 1f). To investigate whether the inhibition of dimeric integrin *α*5*β*1 can influence on the cell adhesion efficacy to FN substrate, integrin *α*5, *β*1, and *α*5*β*1 antibodies (Ab) were pretreated to the single SD‐hESCs to block FN to integrin‐mediated interactions (Figure 1g). After 12 h binding between cells to substrates, cell adhesion efficiency was dramatically decreased in all pretreated groups, with the lowest adhesion observed in integrin *α*5*β*1 Ab‐treated group (1.51 ± 0.35%) compared to the control cells (20.79 ± 1.37%) and other groups (*α*5, 4.19 ± 0.93%; *β*1, 6.06 ± 1.36%). Interestingly, we also confirmed the isolation potential of CD90^+^CD105^+^ cells from even spontaneously differentiated iPSCs on FN substrate (Figure S5, Supporting Information), suggesting this isolation method could be applied to other types of PSCs. These results revealed that FN is a useful substrate to isolate and enrich CD90^+^CD105^+^ cells from SD‐hESCs through the interaction between FN and the integrin *α*5*β*1 in cells.

**Figure 2:**
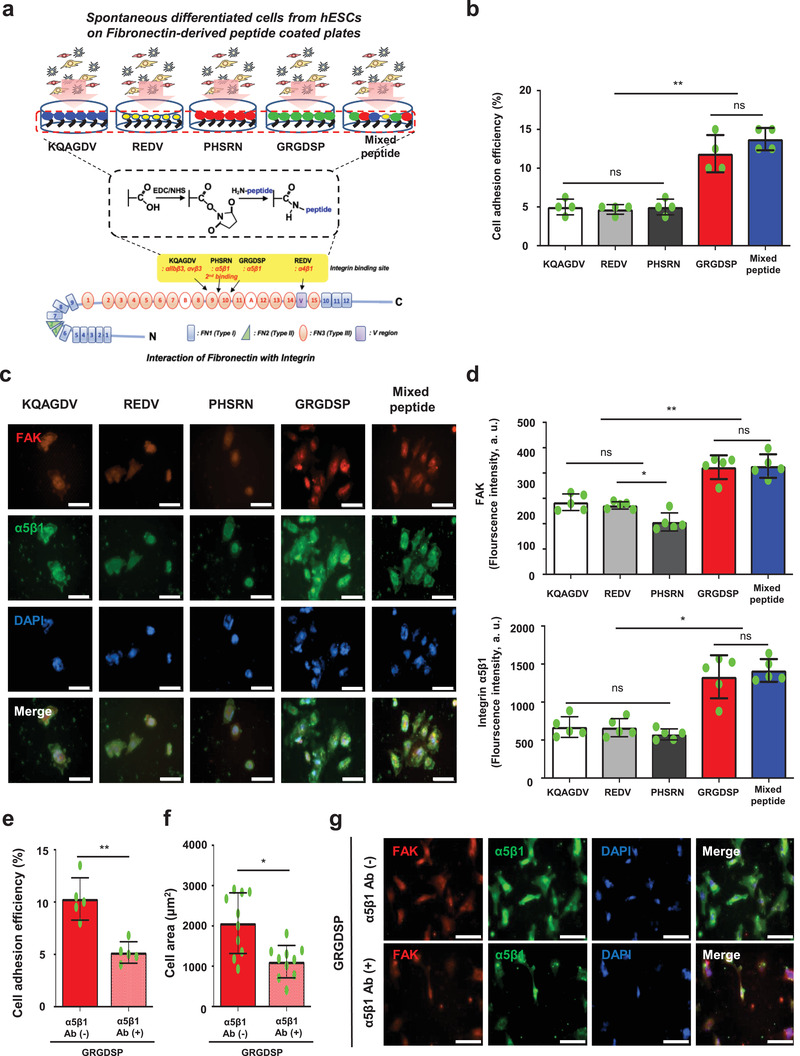
The GRGDSP motif in FN is required for the selection of MSC‐like cells from spontaneous differentiated hESCs via integrin *α*5*β*1. a) Schematic depicting the peptide‐conjugation strategy based on the EDC/NHS complex. b) GRGDSP and mixed peptide‐conjugated group show significantly high level of cell adhesion efficiency. *n* = 4, mean ± s.d., ns, not significant, and ^**^ *P* < 0.01 (one‐way ANOVA). c,d) After 7 days of spontaneous differentiation of hESCs, followed by matrix‐mediated binding for 12 h, the GRGDSP and mixed peptide‐conjugated groups show significantly high levels of FAK and integrin *α*5*β*1 expression with good cell binding and spreading, but not the other peptide‐conjugated groups. Scale bar: 100 µm. *n* = 5, mean ± s.d., ns, not significant, ^*^ *P* < 0.05, and ^**^ *P* < 0.01 (one‐way ANOVA). e,f) Single spontaneously differentiated hESCs subjected to integrin *α*5*β*1 inhibition using antibodies prior to binding to GRGDSP peptide‐conjugated dish. Integrin *α*5*β*1‐inhibited cells exhibited decreased cell adhesion efficiency (*n* = 5) and cell area (*n* = 10). Mean ± s.d., ns, ^*^ *P* < 0.05, and ^**^ *P* < 0.01 (Student's *t*‐test). g) For 12 h on GRGDSP peptide‐conjugated dish, integrin *α*5*β*1‐inhibited cells show significantly low levels of FAK and integrin *α*5*β*1 expression with poor cell binding and spreading. Scale bar: 100 µm.

The studies have reported that FN has multiple functions and contains multiple binding sites, including gelatin, fibrin, glycosaminoglycans, and cell integrin binding.^[^ ^29^ ^]^ Integrin binding ligands of FN have four types of cell binding sites: KQAGDV, REDV, PHSRN, and Gly‐Arg‐Gly‐Asp‐Ser‐Pro (GRGDSP).^[^ ^30^ ^]^ Each KQAGDV, REDV, PHSRN, and GRGDSP peptide has different binding‐integrin dimer subunits (KQAGDV: *α*IIb*β*3 and *α*v*β*3 binding; REDV: *α*4*β*1 binding; PHSRN: *α*5*β*1 second binding; GRGDSP: *α*5*β*1 binding).^[^ ^31^ ^]^ To explore the binding mechanism between FN and CD90^+^CD105^+^ cells from ESCs, we examined which of these peptides in FN are primarily engaged in the adhesion and isolation of CD90^+^CD105^+^ cells from SD‐hESCs by using peptide conjugation (**Figure** **2**a). After 12 h binding of single SD‐hESCs culture on peptide conjugated culture plates, the GRGDSP and mixed peptide‐conjugated groups showed the highest cell adhesion efficiency (GRGDSP: 11.9 ± 1.28%; mixed peptide: 13.8 ± 0.83%) (Figure 2b). In addition, as shown in Figure 2c,d, focal adhesion‐related peptides such as FAK and integrin *α*5*β*1 in cells were significantly higher in both GRGDSP and mixed peptide‐conjugated group with widespread cell morphology compared to the other peptide groups. This result demonstrates that GRGDSP is the main peptide motif in FN responsible for induction of binding MSC‐like cells through integrin *α*5*β*1 interaction. To confirm the direct effect of integrin *α*5*β*1 binding, we coated the culture plates with various FN‐bound dimer integrin Ab, specifically integrin *α*IIb*β*3, *α*v*β*3, *α*4*β*1, and *α*5*β*1 (Figure S6, Supporting Information). After 12 h of spontaneous differentiation of hESCs on each plate, the integrin *α*5*β*1 Ab‐coated group showed the highest cell adhesion. Furthermore, to confirm that the GRGDSP motif is directly associated with integrin *α*5*β*1‐mediated cell binding, we applied integrin *α*5*β*1 Ab to GRGDSP‐conjugated plates, and subsequently cultured SD‐hESCs on the plates. We found that cell adhesion efficacy, cell area, FAK, and integrin *α*5*β*1 were significantly decreased after treatment with the integrin *α*5*β*1 Ab on GRGDSP‐conjugated culture plates (Figure 2e–g). Therefore, we identified GRGDSP as the primary peptide associated with integrin *α*5*β*1 binding interactions compared to the other integrin‐binding peptides in FN and FN‐mimetic mixed peptides. This finding coincides with those of previous studies reporting the GRGDSP motif (natively found in the tenth type III module: FN III10) is the primary recognition site for integrin *α*5*β*1.^[^ ^32^ ^]^ These results confirmed that the GRGDSP motif in FN is indeed required for integrin *α*5*β*1 binding and can be targeted to isolate MSC‐like cells highly expressing integrin *α*5*β*1 from SD‐hESCs.

**Figure 3:**
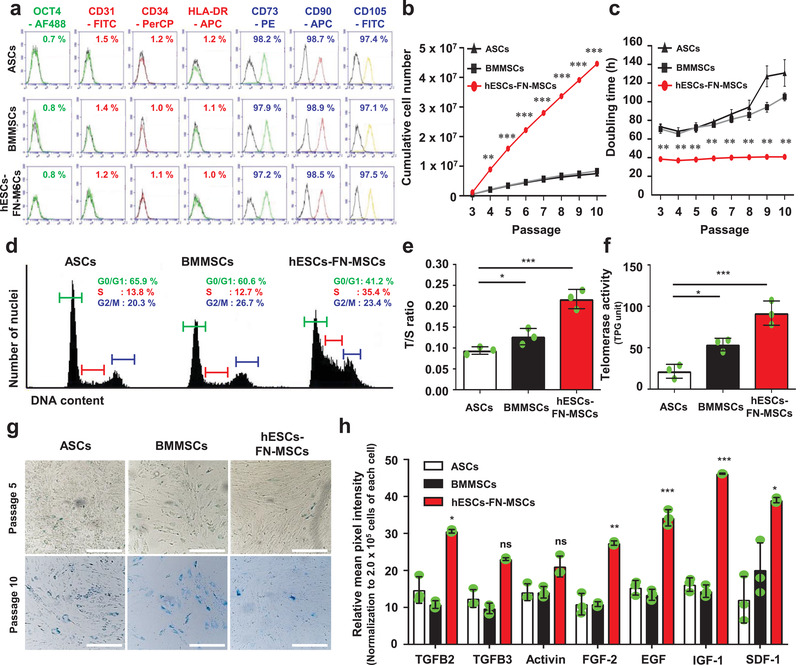
The MSC surface markers of hESC‐FN‐MSCs are similar to both of ASCs and BMMSCs, but hESC‐FN‐MSCs have unique cell behaviors and characteristics. a) Surface antigen profiling with FACS showed that hESC‐FN‐MSCs are similar to ASCs and BMMSCs. OCT4 is a pluripotent marker, while CD73, CD90, and CD105 are positive MSC markers, and CD31, CD34, and HLA‐DR are negative MSC markers. b,c) Upon measuring the cumulative cell number and population doubling time until 10 passages, hESC‐FN‐MSCs showed high proliferative capabilities with short doubling time compared to ASCs and BMMSCs. Interestingly, even at passage 10, hESC‐FN‐MSCs were able to maintain their short doubling time, but ASCs and BMMSCs drastically increased their doubling time. *n* = 4, mean ± s.d., ^**^ *P* < 0.01, and ^***^ *P* < 0.001 versus ASCs and BMMSCs (two‐way ANOVA). d) Representative flow cytometric plot showing the cell cycle analysis of ASCs, BMMSCs, and hESC‐FN‐MSCs at passage 3. A higher number of cells were in the S phase for hESC‐FN‐MSCs compared to both ASCs and BMMSCs. e) Relative telomere length expressed as T/S ratio measured by qRT‐PCR. The relative telomere length of hESC‐FN‐MSCs was longer than those of ASCs and BMMSCs. *n* = 3, mean ± s.d., ^*^ *P* < 0.05, and ^***^ *P* < 0.001 (one‐way ANOVA). f) Telomerase activity in hESC‐FN‐MSCs was higher than that in ASCs and BMMSCs. *n* = 3, mean ± s.d., ^*^ *P* < 0.05, and ^***^ *P* < 0.001 (one‐way ANOVA). g) Comparison of cellular senescence in ASCs, BMMSCs, and hESC‐FN‐MSCs at passages 5 and 10 by SA‐*b*‐gal staining. hESC‐FN‐MSCs at passages 5 and 10 showed a low level of senescence compared with ASCs and BMMSCs. Scale bar: 100 µm. h) The amounts of growth factors and cytokines were significantly higher in hESC‐FN‐MSCs than in ASCs and BMMSCs. *n* = 3, mean ± s.d., ^*^ *P* < 0.05, and ^***^ *P* < 0.001 versus ASCs and BMMSCs (two‐way ANOVA).

For clear understanding, hESC‐FN‐MSCs denoted CD90^+^CD105^+^ cells or MSC‐like cells isolated from SD‐hESCs after 12 h binding selection using FN substrate. After three passages from 12 h binding selection using the FN substrate, the isolated cells showed spindle‐shaped MSC morphology following an epithelial‐to‐mesenchymal transition corresponding to the MSC culture conditions. To define hESC‐FN‐MSCs truly qualify as MSCs, we compared the cell behavior and characteristics between hESC‐FN‐MSCs and representative adult MSCs such as human ASCs and BMMSCs (**Figure** **3**). According to flow cytometry analysis, hESC‐FN‐MSCs showed high expression of positive MSC markers such as CD73, CD90, and CD105, and low expression of hematopoietic markers such as CD31, CD34, and HLA‐DR. Including OCT4 expression, the expression levels of hESC‐FN‐MSCs were comparable to those of ASCs and BMMSCs (Figure 3a). Many previous studies reported that hPSC‐derived MSCs maintain high proliferative capabilities, which is a great advantage in terms of cell quantity for clinical and commercial use.^[^ ^2^, ^33^ ^]^ As shown in Figure 3b,c, hESC‐FN‐MSCs showed high proliferative capabilities, maintaining a constant doubling time until passage 10, whereas ASCs and BMMSCs drastically increased in doubling time with each passage, followed by the retardation of proliferation. According to the cell cycle analysis results (Figure 3d), the population of cells in the S phase, indicating DNA replication in actively proliferating cells, was much higher for hESC‐FN‐MSCs (35.4%) compared to both of ASCs and BMMSCs (13.8% and 12.7%, respectively) without karyotype alteration (Figure S7, Supporting Information). In addition, both telomere length determined by quantitative real‐time polymerase chain reaction (qRT‐PCR) and telomerase activity analysis determined by TRAP assay confirmed the higher proliferative capability of hESC‐FN‐MSCs compared to both of ASCs and BMMSCs (Figure 3e,f). Cellular senescence in ASCs, BMMSCs, and hESC‐FN‐MSCs was analyzed using the staining of senescence‐associated *b*‐galactosidase (SA‐*b*‐gal) activity at passages 5 and 10 (Figure 3g). At passages 5 and 10 of ASCs and BMMSCs exhibited an increased senescence‐associated beta‐galactosidase (SA‐*β*‐gal), while hESC‐FN‐MSCs did not significant difference between passage 5 and passage 10. In addition, senescence‐associated mitochondrial dysfunction‐driven production of reactive oxygen species (ROS) and genes such as *GBL1* and *p21* was mostly lower in hESC‐FN‐MSCs than in ASCs and BMMSCs (Figure S8, Supporting Information). These results indicate that during long‐term culture, hESC‐FN‐MSCs are able to delay the onset of senescence more than adult MSCs such as ASCs and BMMSCs. Previous studies have demonstrated that the therapeutic effects of MSCs largely depend on the secretion of soluble factors such as growth factors and cytokines.^[^ ^34^ ^]^ We quantified the growth factors and cytokines secreted from hESC‐FN‐MSCs and compared them with those secreted from ASCs and BMMSCs (Figure 3h). Transforming growth factor beta 2 and beta 3 (TGF*β*2 and TGF*β*3), activin, fibroblast growth factor (FGF), epidermal growth factor (EGF), insulin‐like growth factor 1 (IGF‐1), and stromal‐derived factor 1 (SDF‐1), which are involved in cell growth, proliferation, and differentiation, were detected in all of the stem cells. However, the amounts of growth factors and cytokines were significantly higher in hESC‐FN‐MSCs than in ASCs and BMMSCs. Collectively, hESC‐FN‐MSCs have MSC characteristics,^[^ ^23^, ^35^ ^]^ and were comparable to those of ASCs and BMMSCs and show better cellular behavior with regard to therapeutic potential such as cell growth, population, cellular senescence status, and therapeutic cytokine secretion.

**Figure 4:**
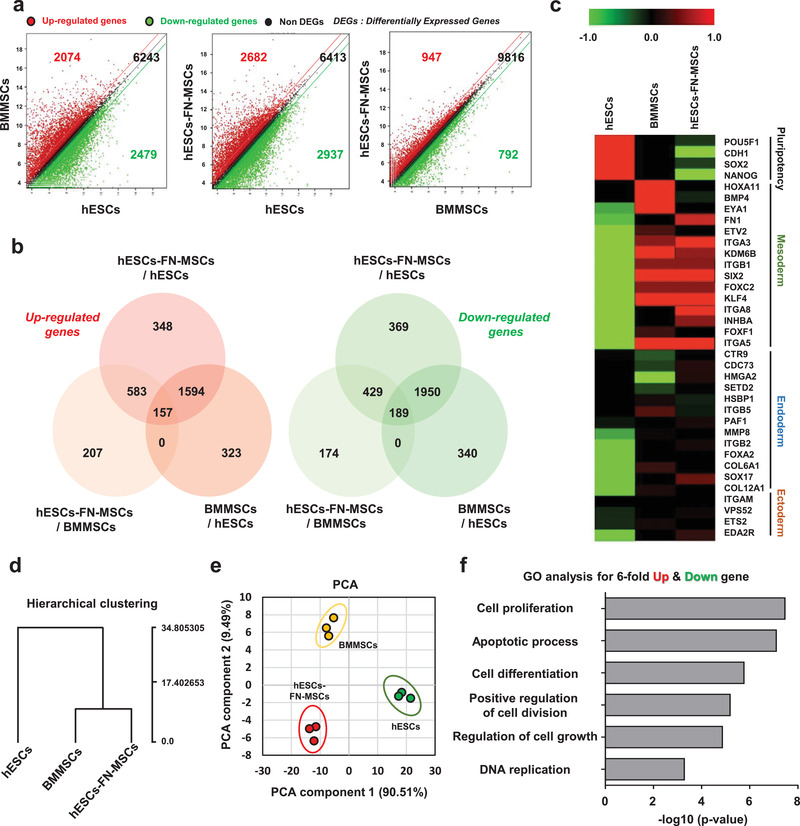
Transcriptomic analysis elucidates the biological similarities between hESC‐FN‐MSCs and BMMSCs. a,b) Scatter plot and Venn diagram of significant differentially expressed genes (SDEs) versus different groups (BMMSCs vs hESCs, hESC‐FN‐MSCs vs hESCs, and hESC‐FN‐MSCs vs BMMSCs). hESC‐FN‐MSCs were more similar to BMMSCs than to hESCs. c) Heat map based on the log_2_ of fragments per kilobase of transcript per million mapped reads (FPKM) for signature genes of pluripotency, mesoderm, endoderm, and ectoderm differentiation for hESCs, BMMSCs, and hESC‐FN‐MSCs samples. A heat map based on the signature genes of mesoderm differentiation showed similar upregulated gene expression patterns between hESC‐FN‐MSCs and BMMSCs. d) Hierarchical clustering of average gene expression profiles in hESCs, BMMSCs, and hESC‐FN‐MSCs. hESC‐FN‐MSCs were very similar to BMMSCs, but showed distinct differences from hESCs. e) PCA of the top 500 high variance genes within hESCs, BMMSCs, and hESC‐FN‐MSCs samples using the first two principal components. Each spot represents a single RNA‐seq. hESC‐FN‐MSCs were more similar to BMMSCs than to hESCs, *n* = 3. f) Significant GO terms for associated biological processes from sixfold up‐ and downregulated genes in hESC‐FN‐MSCs compared to hESCs and BMMSCs. Terms related to biological processes indicate high proliferation and cell replication. Bar charts present the six most significant terms in each category for each cell type sorted by mean −log10 (*P*‐values).

To confirm the similarity and difference of the transcriptome profiles on hESCs, BMMSCs, and hESC‐FN‐MSCs, we investigated the transcriptome profiles of hESCs, BMMSCs, and hESC‐FN‐MSCs by RNA sequencing. Differential expression analysis with CuffDiff^[^ ^36^ ^]^ revealed 10 796, 12 032, and 11 555 modulated genes for BMMSCs versus hESCs, hESC‐FN‐MSCs versus hESCs, and hESC‐FN‐MSCs versus BMMSCs, respectively (fold difference ≥5 and p‐adj <0.05). Both scatter plots and Venn diagram analysis showed that 2074, 2668, and 947 genes were upregulated, while 2479, 2937, and 792 genes were downregulated in BMMSCs versus hESCs, hESC‐FN‐MSCs versus hESCs, and hESC‐FN‐MSCs versus BMMSCs, respectively (**Figure** **4**a,b). The total numbers of differentially expressed genes (both up‐ and downregulated) between hESC‐FN‐MSCs versus BMMSCs were drastically lower than those between hESC‐FN‐MSCs versus hESCs. This implies that hESC‐FN‐MSCs were more similar to BMMSCs than hESCs in terms of the genetic characteristics. To confirm the mesoderm lineage differentiation of hESC‐FN‐MSCs, we investigated the signature genes for pluripotency, mesoderm, endoderm, and ectoderm differentiation in hESC‐FN‐MSCs compared with those in hESCs and BMMSCs (Figure 4c). A heat map showed the signature gene expression for mesodermal differentiation in hESC‐FN‐MSCs was similar to that in BMMSCs but not to that in hESCs. Interestingly, *FN1* and *ITGA5* were highly upregulated in hESC‐FN‐MSCs, indicating cell isolation was achieved through FN‐bound integrin *α*5*β*1 interaction. In addition, as shown in Figure 4d, the hESC‐FN‐MSCs were classified into the same cluster as BMMSCs during hierarchical clustering analysis, but not hESCs. As visualized in the principal component analysis (PCA) plot, hESCs, BMMSCs, and hESC‐FN‐MSCs were independently plotted in distinct groups (Figure 4e). However, the close relationship between hESC‐FN‐MSCs and BMMSCs was also explained by 90.51% of the variance (PCA component 1), while 9.49% of the variance (PCA component 2) was explained by the separation between hESC‐FN‐MSCs and BMMSCs. Furthermore, gene ontology (GO) term enrichment analysis for hESC‐FN‐MSCs versus hESCs and for hESC‐FN‐MSCs versus BMMSCs (Figure 4f) revealed the sixfold up‐ and downregulated genes in hESC‐FN‐MSCs compared to hESCs and BMMSCs were associated with six main biological processes: cell proliferation (GO:0008283), apoptotic processes (GO:0043065), cell differentiation (GO:0030154), positive regulation of cell division (GO:0051781), regulation of cell growth (GO:0001558), and DNA replication (GO:0006260). This implies that all of the six GO terms were highly associated with previous hESC‐FN‐MSCs characteristics such as high proliferation and cell replication. Taken together, the results indicate that the expression of global transcriptome and lineage signature genes between hESC‐FN‐MSCs and BMMSCs was not significantly different, but hESC‐FN‐MSCs are distinctly different from that of hESCs.

**Figure 5:**
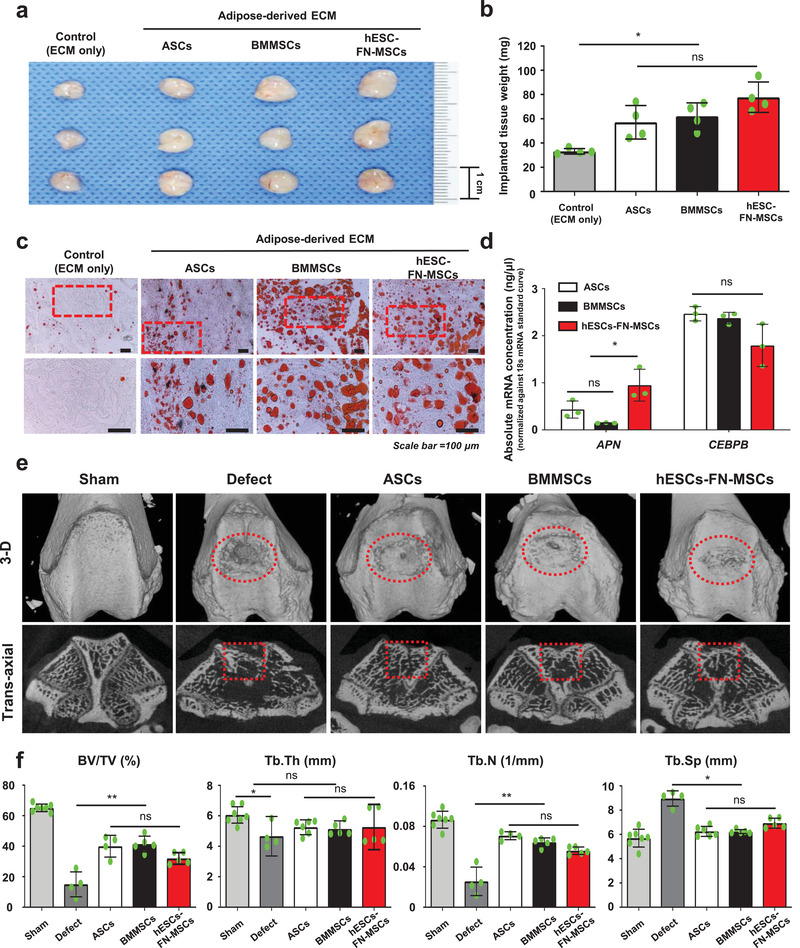
hESC‐FN‐MSCs are capable of in vivo differentiating into cells of multiple lineages. a) Macroscopic appearance of adipose tissue newly formed in nude mice. Human extracellular matrix powders containing ASCs, BMMSCs, and hESC‐FN‐MSCs were implanted into the back of each nude mouse for 5 weeks. The formed tissue in the hESC‐FN‐MSC‐injected group was larger than those in the ASC‐ and BMMSC‐injected groups (*n* = 4–5). b) The weight of adipose tissue formed in the group injected with hESC‐FN‐MSCs was compared with that formed in the groups injected with ASCs and BMMSCs. The formed tissue in the hESC‐FN‐MSC‐injected group was significantly heavier than that in the control (ECM‐only), but was not significantly different compared to the tissue in both the ASC‐ and BMMSC‐injected groups. *n* = 4, mean ± s.d., ns, not significant, and ^*^ *P* < 0.05 (one‐way ANOVA). c) Histologic examination of the formed adipose tissue, stained by Oil red O. The lipid accumulation levels between groups were not significantly different. Scale bar: 100 µm. d) hESC‐FN‐MSC‐injected group exhibited significantly higher levels of gene expression for *APN* than the ASC‐ and BMMSC‐injected groups. However, the expression of *CEBPB* in these groups was not significantly different. *n* = 3, mean ± s.d., ns, not significant, and ^*^ *P* < 0.05 (two‐way ANOVA). e) 3D reconstructed image and cross‐sectional image of micro‐CT at 8 weeks. The cross‐sectional images of the ROI (framed red square) were analyzed by bone histomorphometry, *n* = 7, animal per sham; *n* = 4–6, animal per groups. f) Bone histomorphometry of the 3D bone formation architecture analyzed in an ROI for all groups. The new bone formation in the hESC‐FN‐MSC‐injected group was similar to that in the ASCs and BMMSC‐injected groups. The plots of the parameters: percent bone volume (BV/TV, %), trabecular thickness (Tb.Th, mm), trabecular number (Tb.N, 1/mm), and trabecular separation (Tb.Sp, mm). *n* = 7, animal per sham; *n* = 4–6, animal per groups, mean ± s.d., ns, not significant, ^*^ *P* < 0.05, and ^**^ *P* < 0.01 (one‐way ANOVA).

Before the confirmation of the in vivo multilineage differentiation potential, we simply evaluated the in vitro multilineage differentiation of hESC‐FN‐MSCs by comparison with ASCs and BMMSCs (Figure S9, Supporting Information). The in vitro multilineage differentiation efficacy of hESC‐FN‐MSCs was relatively higher than that of ASCs and BMMSCs based on histological staining and qPCR. Next, we mainly evaluated the in vivo multilineage tissue formation potential (adipose and osteochondral tissue) by administering ASCs, BMMSCs, and hESC‐FN‐MSCs to an animal model. First, we transplanted ASCs, BMMSCs, and hESC‐FN‐MSCs with human adipose tissue‐derived ECM^[^ ^37^ ^]^ into the subcutaneous tissue in the back of mice for 5 weeks. As shown in **Figure** **5**a,b, the artificial adipose‐tissues formed in the ASCs, BMMSCs, and hESC‐FN‐MSC‐transplanted groups were relatively larger and heavier than those formed in the ECM‐transplanted group used as a control. However, the weights of formed tissue among the ASC‐ and BMMSC‐transplanted groups (57.12 ± 13.81 and 62.13 ± 11.16 mg) were not significantly different from that of the hESC‐FN‐MSC‐transplanted group (77.84 ± 12.54 mg). Similarly, Oil Red O staining for the hESC‐FN‐MSC‐transplanted group (Figure 5c) showed no large difference compared to the ASC‐ and BMMSC‐transplanted groups. Consistent with these findings, the expression of *CEBPB*, a representative adipogenic marker, in the hESC‐FN‐MSC‐transplanted group was not statistically different compared with that in both the ASC‐ and BMMSC‐transplanted groups (Figure 5d). However, *APN* expression in the hESC‐FN‐MSC‐transplanted group was significantly different. Next, to evaluate possible improvement in terms of osteochondral tissue regeneration, we transplanted ASCs, BMMSCs, and hESC‐FN‐MSCs with 2% hyaluronic acid into an osteochondral defect in rats (Figure 5e,f). 3D microcomputed tomography (micro‐CT) evaluation of complete reconstructions and transaxial sectional cuts to 8 weeks post‐transplantation revealed a large volume of new bone formation in the ASC‐, BMMSC‐ and hESC‐FN‐MSC‐transplanted groups. Interestingly, however, many parameters such as percent bone volume (BV/TV, %), trabecular thickness (Tb.Th), trabecular number (Tb.N), and trabecular separation (Tb.Sp) did not show significant differences among the ASC‐, BMMSC‐, and hESC‐FN‐MSC‐transplanted groups. Importantly, the crucial issues in hESC‐ or hiPSC‐based therapy are teratoma formation due to residual undifferentiated ESCs.^[^ ^38^ ^]^ We confirmed that the hESC‐FN‐MSCs produced no teratoma in severe combined immunodeficient (SCID) mice after 10 weeks of transplantation (Figure S10, Supporting Information). Thus, these results confirm that hESC‐FN‐MSCs have in vivo tissue formation potential comparable to that of ASCs and BMMSCs.

Owing to the evolution of new biotechnologies and a better understanding of various principles of life science, many studies have reported that FN mediates various cellular interactions with the ECM and has a crucial role in migration, cell adhesion, growth, and differentiation.^[^ ^39^, ^40^, ^41^ ^]^ Also, many studies have reported that integrins, a major family of adhesion molecules, facilitate the adhesion of cells to surrounding substrates^[^ ^42^ ^]^ and subsequently modulate a variety of cellular responses, including cell attachment, proliferation, induction of gene expression, cell spreading, suppression of apoptosis, and the initiation of differentiation.^[^ ^43^ ^]^ In spite of that; to date, few studies have been conducted regarding the interaction of MSCs and integrin *α*5*β*1‐mediated FN binding. For instance, Veevers‐Lowe et al. found that the adhesion to FN through integrin *α*5*β*1 specifically induced MSC migration by activating PDGFR‐*β* signaling.^[^ ^39^ ^]^ Other papers reported that FN promotes self‐renewal in mouse ESCs.^[^ ^40^ ^]^ Integrin *α*5*β*1 and its FN ligand play critical roles in blood vessel development in mouse embryos and within EBs differentiated from ESCs.^[^ ^41^ ^]^ However, there has been no methodological study of MSC isolation from PSCs using integrin *α*5*β*1‐mediated cell to FN binding.

In summary, to overcome the limitations associated with the adult MSCs and conventional methods for MSC isolation from PSCs, we developed a new method to physically expose cells to certain types of substrates to achieve spontaneous differentiation from ESCs. In other words, we demonstrated for the first time that isolating hMSCs based on specific binding interactions between MSCs and FN is superior to conventional methods due to no requirements for: 1) MSC sorting with flow cytometry, 2) long‐term cultivation, and 3) complicated combination treatment with both growth factors and cytokines. More importantly, with our novel method, the generation of MSCs from ESCs can be completed in 20 days, compared to >30 days using conventional methods.

## 3 Conclusion

We have demonstrated an efficient and simplified process to isolate homogeneous MSCs from SD‐ESCs via integrin *α*5*β*1‐mediated interactions with FN. Efficient isolation of MSCs out of this heterogeneous mixture of differentiating ESCs is technically challenging. But, these SD‐ESCs are a type of ESCs capable of differentiating into all the three different germ layers including MSCs. We have focused on the fact that FN is known to specifically bind to integrin *α*5*β*1, which is highly expressed on MSCs. This unique binding is sufficient to isolate only MSCs from the heterogeneous population of SD‐ESCs. Additional 9 days culture of these cells enables to obtain >99% pure MSCs without complicated sorting steps. The final goal is to validate our new method as an innovative versatile technology to isolate MSCs from ESCs with broad applicability for stem cell‐therapeutics in regenerative medicine.

## 4 Experimental Section

##### hESC Culture

Undifferentiated hESCs (H9, WiCell Research Institute, female, passages 34–50) were cultured on mitotically inactivated mouse embryonic fibroblasts (MEFs, ATCC) and maintained in Dulbecco's modified Eagle medium (DMEM)/F12 medium (Invitrogen) supplemented with 20% serum replacement (SR, Invitrogen), 1% nonessential amino acids (NEAA, Invitrogen), 1% penicillin–streptomycin (P/S, Invitrogen), 0.1 × 10^−3^ m *b*‐mercaptoethanol (Invitrogen), and 4 ng mL^−1^ basic fibroblast growth factor (bFGF, Invitrogen). The MEFs were seeded on 35 mm culture dishes coated with 0.1% porcine gelatin (Sigma) and cultured for 24 h. Following adhesion of the MEFs to the 35 mm culture dishes, hESC colonies were mechanically segregated using a Pasteur pipette without any enzyme treatment and then replated on freshly prepared MEF feeder. After 48 h incubation, the medium was refreshed every 24 h.

##### Adult Stem Cells Culture

Human ASCs (ATCC) and BMMSCs (ATCC) were incubated in DMEM (Gibco BRL) supplemented with 10% fetal bovine serum (FBS; Gibco BRL) and 100 units mL^−1^ penicillin (Gibco BRL) in humidified air with 5% CO_2_ at 37 °C. The media was changed every 2 days and the cells were passaged at 80% confluency.

##### Matrix Coating on Culture Dishes

To prepare cells for matrix‐mediated binding, the cells were seeded on 35 mm tissue culture dishes coated with gelatin (Sigma), PLL (Sigma), and FN (Peprotech). Dishes were coated by covering with a thin layer of 1 mL of 1 mg mL^−1^ gelatin, 100 µg mL^−1^ PLL, or 20 µg mL^−1^ FN diluted in 1× phosphate‐buffered saline (PBS, Gibco Invitrogen) at pH 7.4^[^ ^44^ ^]^ and incubating at room temperature (RT) for at least 1 h. The unbound substrate was aspirated and air‐dried at RT for at least 45 min. The coated dishes were used either immediately or stored at 4 °C for 24–48 h before use. An uncoated culture dish was used as the control surface (None, tissue culture treated).

##### Matrix‐Mediated Binding Separation of MSCs from Spontaneously Differentiated hESCs

Figure 1a presents the overall procedures for the separation of MSCs from hESCs. Briefly, small clumps of hESCs were transferred into Matrigel (BD Biosciences)‐coated dishes in E8 feeder‐free media (Invitrogen) and stabilized under humidified air with 5% CO_2_ at 37 °C. The medium was refreshed daily and maintained until the hESC colonies reached 70% confluency. For spontaneous differentiation of hESCs, the cells were incubated in spontaneous differentiation medium comprising DMEM/F12 medium supplemented with 10% FBS (Highclone), 1% NEAA, and 1% P/S for 7 days. During these 7 days, the cells were treated with 1% DMSO (Sigma) for 12 h during the early stage of spontaneous differentiation and with 10 × 10^−9^ m YM‐155 (Calbiochem) for 1 day before the end of spontaneous differentiation. To separate the MSC‐like cells from the spontaneously differentiated cells via matrix‐mediated binding, the cells were dissociated into single cells and seeded onto uncoated, gelatin‐coated, PLL‐coated, and FN‐coated dishes at a density of 5 × 10^4^ cells cm^−2^, as indicated above. Uncoated culture dishes were used as the control. After incubation for 12 h at 37 °C, nonadherent cells were removed by rinsing with PBS. Adherent cells on each matrix were cultured for 4 days and visualized using differential interference contrast (DIC) microscopy. The medium was refreshed every 2 days. These adherent cells were maintained and subcultured every 3–4 days until the third passage.

##### Cell Adhesion Assay

Cells were dissociated into single cells using 0.05% trypsin/ethylenediaminetetraacetic acid (EDTA), seeded onto surfaces conditioned with various matrix coatings at a density of 5 × 10^4^ cells cm^−2^, and allowed to adhere for 12 h at 37 °C. Afterward, nonadherent cells were gently removed by washing with PBS, and adherent cells under each condition were counted using a hemocytometer. The cell adhesion efficiency was calculated with the following equation: (number of attached cells/number of initial cells) × 100.

##### Cell Proliferation and Population Doubling Time Assay

To determine the cumulative cell numbers from passage 3 to passage 10, the cells at each passage were trypsinized from individual wells, transferred to low‐glucose (LG) DMEM containing 10% FBS to neutralize the trypsin, and counted with a standard hemocytometer. To evaluate the population‐doubling time (PDT), human ASCs (ATCC), BMMSCs (ATCC), and hESC‐FN‐MSCs at passage 3 were counted and cultured at a starting number of 5 × 10^3^ cells per well in 24‐well plates. At 80% confluency, the cells were trypsinized, counted, and replated. PDT evaluation was repeated three times starting from passage 3 for each group of cells and the average cell number was used for the final calculations. This process was continued up to passage 10 (minimum 28 days) and the in vitro doubling time was calculated using the exponential curve equation.

##### Immunostaining

Samples were fixed with 4% paraformaldehyde (Sigma), permeabilized with Triton X‐100 (Sigma), and blocked with a mixture of bovine serum albumin (BSA, Sigma) and either goat or donkey serum, depending on the species of secondary antibody used. Samples were incubated with primary antibodies (Antibeta Actin (Santa Cruz, 1:200), Anti‐Vinculin (Millipore Sigma, 1:200), Anti‐FAK (Millipore Sigma, 1:200), and Anti‐Integrin *α*5*β*1 (Millipore Sigma, 1:200)) overnight at 4 °C and washed. The samples were subsequently stained with fluorescently labeled secondary antibodies (Alexa Fluor‐488 or 594 conjugated goat antirabbit (Abcam, 1:400), Alexa Fluor‐488 or 594 conjugated goat antimouse (Abcam, 1:400)) for 30 min at RT. 4′,6‐diamidino‐2‐phenylindole dihydrochloride (DAPI, Sigma) was applied as a nuclear counterstain for 5 min at RT. The samples were washed and mounted for imaging with a confocal microscope (LSM 710, Zeiss) and fluorescence microscope (IX71 inverted microscope, Olympus). The relative surface area of coverage for stains was quantified with the ImageJ software (NIH, version 1.25p).

##### FACS Analysis

Cell surface antigens on cells were evaluated with FACS. The cells were dissociated with 0.05% trypsin/EDTA (Highclone), washed with PBS, fixed with 4% paraformaldehyde, permeabilized with Triton X‐100, and blocked with a BSA mixture. The samples were then stained with antibodies against human octamer‐binding transcription factor 4 (OCT4; R&D systems, 1:200), stage specific embryonic antigen 1 (SSEA1; R&D systems, 1:200), cluster of differentiation 31 (CD31‐FITC; Miltenyi Biotec, 1:100), CD34 (CD34‐PerCP, BioLegend, 1:100), human leukocyte antigen ‐ DR isotype (HLA‐DR, HLA‐DR‐APC, BioLegend, 1:100), CD73 (CD73‐PE, BioLegend, 1:100), CD90 (CD90‐APC, BioLegend, 1:200), CD105 (CD105‐FITC, BioLegend, 1:150), integrin *α*5 (Santa Cruz, 1:200), integrin *α*11 (Abcam, 1:200), integrin *β*1(Abcam, 1:200), and integrin *β*5 (BioLegend, 1:200) for 30 min or 1 h at 4 °C. Samples were subsequently stained with fluorescently labeled secondary antibodies (Alexa Fluor‐488 or 594 conjugated goat antirabbit (Abcam, 1:400), Alexa Fluor‐488 or 594 conjugated goat antimouse (Abcam, 1:400)) for 30 min at 4 °C. The corresponding mouse/rabbit isotype antibodies (Abcam, 1:200) were used as controls. Cell immunotypes were determined with the Accuri C6 flow cytometer (BD Biosciences) and the percentage of expressed cell surface antigens was calculated for 10 000 gated‐cell events.

##### RNA Isolation and qRT‐PCR

Total RNA was isolated using TRIzol reagent (Invitrogen) according to the manufacturer's protocol. mRNA was reverse‐transcribed into complementary DNA (cDNA) using TOPscriptTM cDNA Synthesis kit (Enzynomics, South Korea). Quantitative PCR analysis was performed using the Power SYBR Green PCR Master Mix (Applied Biosystems) with a StepOnePlus Real‐Time PCR System (Applied Biosystems) according to the manufacturer's instructions. Target gene expression was normalized to the glyceraldehyde‐3‐phosphate dehydrogenase (GAPDH) gene for quantification. Primer sequences used for qRT‐PCR analysis are shown in **Table** **1**.

**Table 1:** Primer sequences for qRT‐PCR

Gene	Forward primer (5′‐3′)	Reverse primer (5′‐3′)
*ITGA1*	AAT TGG CTC TAG TCA CCA TTG TT	CAA ATG AAG CTG CTG ACT GGT
*ITGA4*	GAT GAA AAT GAG CCT GAA ACG	GCC ATA CTA TTG CCA GTG TTG A
*ITGA5*	TGC AGT GTG AGG CTG TGT ACA	GTG GCC ACC TGA CGC TCT
*ITGA7*	GAC GAC GGT CCC TAC GAG	GAC CTT TCC CCG AGT CAA TAG
*ITGA10*	GTG TGG ATG CTT CAT TCC AG	GCC ATC CAA GAC AAT GAC AA
*ITGA11*	CCA ACC CCA AGG ACA ACA	CTC CCA CAC TCA TGA GAC CA
*ITGAV*	GCA CCC TCC TTC TGA TCC T	GAG GAC CTG CCC TCC TTC
*ITGB1*	GAA GGG TTG CCC TCC AGA	GCT TGA GCT TCT CTG CTG TT
*ITGB2*	CAG CAA TGT GGT CCA TCT CA	GAG GGC GTT GTG ATC CAG
*ITGB3*	CGC TAA ATT TGA GGA AGA ACG	GAA GGT AGA CGT GGC CTC TTT
*ITGB5*	GGG AGT TTG CAA AGT TTC AGA G	TGT GCG TGG AGA TAG GCT TT
*ITGB8*	GCA TTA TGT CGA CCA AAC TTC A	GCA ACC CAA TCA AGA ATG TAA CT
*APN*	ACT GCA GTC TGT GGT TCT GA	CAT GAC CGG GCA GAG CTA AT
*CEBPB*	GCA AGA GCC GCG ACA AG	GGC TCG GGC AGC TGC TT
*GAPDH*	ACA TCG CTC AGA CAC CAT G	TGT AGT TGA GGT CAA TGA AGG G

##### Gene Expression Quantified via the Relative Standard Curve Method

The concentrations of mRNA in each type of groups were measured via the relative standard curve method.^[^ ^45^ ^]^ A series of tenfold dilution of each cDNA with known concentrations (1000, 100, 10, 1, 0.1, and 0.01 ng) were first made and were then used to amplify the 18S rRNA endogenous control. In a semilogarithmic graph with base 10, the threshold cycle (*C*_T_) values were plotted against their respective dilution factors to create a standard curve and fitted in a straight line to generate a linear regression equation. Diluted samples of each cDNA were then used to amplify the different target integrins in triplicates and their concentrations were determined using the equation *N* = $10^{\frac{C_{T}-b}{m}}$, where *C*_T_ = threshold value, *b* = *Y*‐intercept, and *m* = slope, from the 18S rRNA standard curve. The primer sequences of 18S rRNA were forward primer, GTA ACC CGT TGA ACC CCA TT; reverse primer, CCA TCC AAT CGG TAG TAG CG.

##### Integrin *α*5*β*1 Receptor Blocking

FN‐mediated cells in the first 12 h were pretreated with 10 µg mL^−1^ Anti‐Integrin *α*5 (Abcam), Anti‐Integrin *β*1 (Abcam), Anti‐Integrin *α*5*β*1 antibody (Millipore), or noninhibitory isotype control antibody (Millipore) for 30 min at 37 °C before washing in PBS. The cells (2 × 10^4^ cells cm^−2^) were seeded in FN‐coated dishes for 1 h at 37 °C. After removal of nonadherent cells by washing with PBS, adherent cells were harvested with trypsin and quantified in triplicates with a hemocytometer. In addition, the area of adherent cells was quantified with the ImageJ software (NIH, version 1.25p).

##### FN‐Derived Cell Binding Peptide Graft

To investigate which cell adhesion binding motif in FN can affect the interaction with integrin *α*5*β*1 in hESC‐derived spontaneously differentiated cells at 7 days, four peptides derived from FN (KQAGDV, REDV, PHSRN, and GRGDSP) were grafted onto culture plates. Briefly, plastic 24‐ and 6‐well carboxyl‐derivatized plates (Costar Corporation) were reacted using a solution of 1‐ethyl‐3‐(3‐dimethylaminopropyl) carbodiimide (EDC) and *N*‐hydroxysulfosuccinimide (NHS) as coupling reagents in PBS at pH 7.4 for 2 h at RT. Plates containing activated carboxylic groups were reacted with a solution containing 0.05% (w/v) soluble peptides (GGG‐KQAGDV, GGG‐GREDV, GGG‐PHSRN, and GGG‐GRGDSP obtained from BioActs (Incheon, South Korea)) in PBS at pH 10.5 for 24 h at 4 °C. The solution containing unreacted peptides was withdrawn and the plates were rinsed three times with distilled water. The aqueous solution was then removed and the membranes were dried in an oven at 37 °C.

##### Cell Cycle Analysis

The cell cycle distribution was examined by measuring the DNA content of nuclei labeled with propidium iodide (PI). ASCs, BMMSCs, and hESC‐FN‐MSCs were harvested at passage 3 by centrifugation, washed with 1 mL cold PBS, centrifuged, and fixed in 70% cold ethanol at 4 °C for 24 h. Subsequently, the cells were washed twice and treated with RNase A (20 µg µL^−1^) and PI (20 µg µL^−1^) for 30 min at 37 °C in the dark. Afterward, cell cycle distribution analysis was performed using flow cytometry and the percentages of cells at the G1, S, and G2/M phases were calculated with FlowJo software (version 10.4.2).

##### Telomere Length Measurement

ASCs, BMMSCs, and hESC‐FN‐MSCs at passage 3 were collected at enrollment and stored at −80 °C until genomic DNA extraction. Genomic DNA was extracted directly from samples using standard procedures. Genomic DNA was then used as a template for PCR‐based measurement of relative telomere length according to previously published protocols.^[^ ^46^ ^]^ The assay was performed using 100–200 ng of template DNA in 1 mL aliquots for triplicate PCR amplification per sample per plate. The cycle threshold was transformed into nanograms of DNA based on a standard curve. This quantitative assay determines the amount of telomeric DNA (T) relative to the amount of single‐copy control gene (36B4) DNA (S) and then calculates a T/S ratio. The final telomere primer concentrations were tel 1, 270 × 10^−9^ m and tel 2, 900 × 10^−9^ m. The final 36B4 (single‐copy gene) primer concentrations were 36B4u, 300 × 10^−9^ m and 36B4d, 500 × 10^−9^ m. The primer sequences (written 50/30) were tel 1, GGT TTT TGA GGG TGA GGG TGA GGG TGA GGG TGA GGG T; tel 2, TCC CGA CTA TCC CTA TCC CTA TCC CTA TCC CTA TCC CTA; 36B4u, CAG CAA GTG GGA AGG TGT AAT CC; and 36B4d, CCC ATT CTA TCA TCA ACG GGT ACA A. The thermal cycling profile for both amplicons began with 95 °C incubation for 10 min to activate the SYBR supermix. For telomere PCR, there followed 18 cycles of 95 °C for 15 s and 54 °C for 2 min. For 36B4 PCR, there followed 30 cycles of 95 °C for 15 s and 58 °C for 1 min. The company's software package (Exicycler 96; Bioneer, South Korea) was used to generate a standard curve for each plate and to determine the dilution factors for the standards corresponding to the amounts of T and S in each sample.

##### Telomerase Repeated Amplification Protocol (TRAP) Assay

Telomerase activity was measured using a TRAP assay (TRAPEZE XL telomerase detection kit) following the method proposed by Kim et al.^[^ ^47^ ^]^ The supernatant was poured into microtubes, frozen in liquid nitrogen, and stored at −80 °C. Extracts containing 1.5–2.0 µg µL^−1^ protein were used for telomerase assays.

##### Cell Senescence

Senescence‐associated *b*‐galactosidase (SA‐*b*‐gal) activity at pH 6.0 was detected histochemically in subconfluent cultures for 3 or 4 days using the Senescence *β*‐galactosidase Staining kit (Cell Signaling Technology).

##### Cytokine Expression Profiling

ASCs, BMMSCs, and hESC‐FN‐MSCs at passage 3 were cultured for 3 days, after which fresh medium was added. The cells were conditioned for 48 h and laden onto a human cytokine antibody array (Human Cytokine Array C6, RayBiotech Inc), processed, and detected according to the manufacturer's protocol. Immunoreactivity was detected using the ChemiDocTM XRS+ detection system (BIORAD iNtRON Biotechnology, Seoul, South Korea). The signal densities for each protein were semiquantitatively analyzed using Image Lab software (Bio‐Rad) and normalized to 2 × 10^4^ cells for each group.

##### Library Preparation and RNA Sequencing

A library was constructed using the SENSE mRNA‐Seq Library Prep Kit (Lexogen) according to the manufacturer's instructions. Briefly, 2 µg total RNA was prepared and incubated with magnetic beads decorated with oligo‐dT and all RNAs except mRNAs were removed. Library production was initiated by the random hybridization of starter/stopper heterodimers to the poly(A) RNA still bound to the magnetic beads. These starter/stopper heterodimers contained Illumina‐compatible linker sequences. A single‐tube reverse transcription and ligation reaction extended the starter to the next hybridized heterodimer, where the newly synthesized cDNA insert was ligated to the stopper. Second‐strand synthesis was performed to release the library from the beads, and the library was amplified. Barcodes were introduced when the library was amplified. High‐throughput sequencing was performed as paired‐end 100 sequencing using HiSeq 2000 (Illumina, Inc.). RNA‐Seq reads were mapped using the TopHat software tool to obtain an alignment file, which was used for assembling transcripts, estimating their abundances, and detecting the differential expression of genes or isoforms using cufflinks. Gene classification was based on searches of BioCarta (http://www.biocarta.com/), GenMAPP (http://www.genmapp.org/), DAVID (http://david.abcc.ncifcrf.gov/), and Medline databases (http://www.ncbi.nlm.nih.gov/). Library preparation and RNA sequencing were performed with NGS services provided by Ebiogen Inc. (Seoul, South Korea).

##### In Vivo Adipose Tissue Formation of hESC‐FN‐MSCs via Human Adipose‐ECM Powders

Animal surgeries were performed according to protocols approved by the CHA University Institutional Animal Care and Use Committee guidelines for the care and use of laboratory animals (Approval number # IACUC180036). The human ECM powders were obtained as described previously.^[^ ^48^ ^]^ Suspensions of human ECM powders with PBS (200 µL) or human ECM powders with PBS (200 µl) containing ASCs, BMMSCs, or hESC‐FN‐MSCs at passage 3 (1 × 10^6^ cells) were injected subcutaneously into the backs of 6 week old female mice (C57 BL/6, Orientbio, South Korea) using an 18‐gauge needle. At 5 weeks after the injections, the grafts were explanted, weighed, and fixed with 4% paraformaldehyde. Five mice were analyzed from each experimental group.

##### Histological Analysis

The grafts were assessed by Oil Red O staining of frozen sections. The tissue samples were fixed in 10% sucrose (Sigma). After being embedded in optimal cutting temperature (OCT) compound (Tissue‐Tek O.C.T. Compound, Sakura Finetek, Tokyo, Japan), the samples were frozen at −80 °C. The frozen samples were sliced into 10 mm sections using a cryostat, washed with distilled water and 30% isopropanol to remove the OCT compound, and then stained with the Oil Red O working solutions (Sigma).

##### In Vivo Osteochondral Defect Model

Animal surgeries were performed according to protocols approved by the CHA University Institutional Animal Care and Use Committee for the care and use of laboratory animals (Approval number # IACUC180016). Healthy male Sprague Dawley rats (12 weeks old and weighing 300–350 g) were used for the study. Animals were anesthetized with a mixture of tiletamine hydrochloride, zolazepam hydrochloride (Zoletil, 50 mg kg^−1^, Virbac Laboratories, Carros, France), and xylazine (Rompun, 10 mg kg^−1^, Bayer, Seoul, South Korea). During surgery, each animal was administered an intraperitoneal injection of normal saline to account for fluid loss. Osteochondral defect generation and subsequent transplantation of ASCs, BMMSCs, and hESC‐FN‐MSCs (1 × 10^7^ cells per site, respectively) in rats were performed as follows: a lateral parapatellar longitudinal incision was made to expose the knee joint during surgery. The synovial capsule was incised and the trochlear groove was exposed after medial luxation of the patella. With the knee maximally flexed, a defect 2 mm in diameter and 2 mm in depth was created in the center of the groove using a dental drill. All debris was removed from the defect with a curette and irrigation. Depending on the experimental group, the defect was left untreated or treated with ASCs, BMMSCs, and hESC‐FN‐MSCs in 2% hyaluronic acid (Sigma). The patella was physically relocated and the joint capsule and subcutaneous tissue were closed.

##### Microcomputed Tomography Analysis

The microstructural morphology of the lumbar spines was evaluated using a SkyScan‐1076 micro‐CT device (SkyScan, Kontich, Belgium) at 12 weeks after implantation. The X‐ray source was set to a voxel size of 18 mm at 40 keV and 250 mA. The exposure time was 520 ms with a frame average of 3. X‐ray beam filtration with 1 mm aluminum was used. Data were recorded at rotation step intervals of 0.4° until 180°. Image slices were reconstructed using the NRecon software (Skyscan) based on the Feldkamp algorithm and by applying a correction for the beam. For bone volume (BV) and density calculation, the new bone mass was isolated from the native bone through a manually drawn region of interest (ROI). The outline of the ROIs was manually drawn using CT‐Analyser 3D data analysis software (Skyscan) and care was taken not to select outgrowing mineralized osteophytes. To quantify the density of bone formed within each new mass, the tissue volume (TV) of the mass, trabecular BV within the mass, and percent BV (BV/TV, %) were calculated. In addition, trabecular thickness (Tb.Th mm), trabecular number (Tb.N 1/mm), and trabecular separation (Tb.Sp mm) were calculated.

##### Statistical Analysis

At least three independent sets of experiments for each condition were performed in triplicate. Statistical analysis was performed in Graphpad Prism 6. All data were presented in mean ± SD. Two‐tailed Student's *t*‐tests were used for comparisons between two experimental groups. All data were analyzed with one‐way ANOVA with Bonferroni post hoc tests or two‐way ANOVA with Bonferroni post hoc tests. Independent biological replicates were used to determine *n* values. Statistical significance threshold of each test was set at *P* < 0.05: ns = not significant, *P* > 0.05; ^*^ *P* < 0.05; ^**^ *P* < 0.01; ^***^ *P* < 0.001.

## Conflict of Interest

The authors declare no conflict of interest.

## Supporting information

Supporting Information
