# Supplementary material for: Efficient Isolation and Enrichment of Mesenchymal Stem Cells from Human Embryonic Stem Cells by Utilizing the Interaction between Integrin α5β1 and Fibronectin
Source: Adv Sci (Weinh). 2020 Jul 19;7(17):2001365. doi: 10.1002/advs.202001365 (PMC7507081; doi:10.1002/advs.202001365)
Supplement: Supplementary file 1 — Supporting Information [file ADVS-7-2001365-s001.pdf]

Copyright WILEY-VCH Verlag GmbH & Co. KGaA, 69469 Weinheim, Germany, 2019.

Supporting Information

Efficient isolation and enrichment of mesenchymal stem cells from human embryonic stem cells by utilizing the interaction between integrin α5β1 and fibronectin

*Byung-Hyun Cha, Jin-Su Kim, Alvin Bello, Geun-Hui Lee, Do-Hyun Kim, Byoung Ju Kim, Yoshie Arai, Bogyu Choi, Hansoo Park and Soo-Hong Lee**

Experimental Section

Induced Pluripotent Stem Cell (hiPSCs) Culture: The hiPSCs (hFSiPS1, Korea National Institute of Health, passage 45-50) were cultured on mitotically inactivated mouse embryonic fibroblasts (MEFs) and maintained in Dulbecco's Modified Eagle's Medium (DMEM)/F12 medium (Gibco Invitrogen) supplemented with 20% serum replacement (SR, Gibco Invitrogen), 1% nonessential amino acids (NEAA, Gibco Invitrogen), 1% penicillin-streptomycin (P/S, Gibco Invitrogen), 0.1 mM b-mercaptoethanol (Gibco Invitrogen), and 4 ng ml^{-1} basic fibroblast growth factor (bFGF, Gibco Invitrogen). The MEFs were seeded on 35-mm culture dishes coated with 0.1% porcine gelatin and cultured for 24 hr. Following adhesion of the MEFs to the 35-mm culture dishes, hESCs colonies were mechanically segregated using a Pasteur pipette without any enzyme treatment and then replated on freshly prepared MEFs feeder. After 48 hr incubation, the medium was refreshed every 24 hr.

Matrix-mediated Binding Separation of MSCs from Spontaneously Differentiated hiPSCs: As followed by Figure 1a, small clumps of hiPSCs were transferred into Matrigel (BD Biosciences)-coated dishes in E8 feeder-free media (Invitrogen) and stabilized under humidified air with 5% CO_2 at 37 °C. The medium was refreshed daily and maintained until the hiPSCs colonies reached 70 % confluency. For spontaneous differentiation of hiPSCs, the cells were incubated in spontaneous differentiation medium comprised of DMEM/F12

supplemented with 10% FBS, 1% NEAA, and 1% P/S for 7 days. During this period, the cells were treated with 1% (v/v) DMSO for 12 hr in the early stage of spontaneous differentiation, and 10 nM YM-155 (Calbiochem) for 1 day before the end of spontaneous differentiation. To separate the MSC-like cells from the spontaneously differentiated cells *via* matrix-mediated binding, the cells were dissociated into single cells and seeded onto uncoated (none), gelatin, PLL, and FN-coated dishes at a density of 5×10^4 cells cm^{-2}, as indicated above. Uncoated culture dishes were used as the control. After matrix-mediated binding selection for 6, 12 and 24 hr at 37 °C, non-adherent cells were removed by rinsing with PBS.

Fluorescence-Activated Cell Sorting (FACS) Analysis: Cell surface antigens on cells were evaluated with FACS. The cells were dissociated with 0.05% trypsin/EDTA (Highclone), washed with PBS, fixed with 4% paraformaldehyde, permeabilized with Triton X-100, and blocked with a mixture of BSA. The samples were then stained with antibodies against human integrin α5 (Santa Cruz, 1:200), integrin α11 (Abcam, 1:200), integrin β1(Abcam, 1:200), and integrin β5 (BioLegend, 1:200) for 30 min or 1 hr at 4 °C. Samples were subsequently stained with fluorescently labeled secondary antibodies (Alexa Fluor-488 or 594 conjugated goat anti-rabbit (Abcam, 1:400), Alexa Fluor-488 or 594 conjugated goat anti-mouse (Abcam, 1:400)) for 30 min at 4 °C. The corresponding mouse/rabbit isotype antibodies were used as controls. Cell immunotypes were determined with the Accuri C6 flow cytometer (BD Biosciences) and the percentage of expressed cell surface antigen was calculated for 10,000 gated-cell events.

RNA Isolation and Quantitative Real-Time Polymerase Chain Reaction (qRT-PCR): Total RNA was isolated using TRIzol reagent (Invitrogen) according to the manufacturer's protocol. mRNA was reverse-transcribed into complementary DNA (cDNA) using the TOPscript™ cDNA Synthesis kit (Enzynomics, South Korea). Quantitative PCR analysis was performed using the Power SYBR Green PCR Master Mix (Applied Biosystems) with a StepOnePlus

Real-Time PCR System (Applied Biosystems) according to the instructions. Target gene expression was normalized to the glyceraldehyde-3-phosphate dehydrogenase (GAPDH) gene for quantification. Primer sequences used for qRT-PCR analysis are shown in Table S1. Figure S1a and S4a, Supporting Information shows the absolute levels of each mRNAs calculated using the 18s rRNA gene expression standard curve method[1], as described previously.

Cell Panning: To determine whether the attachment of spontaneously differentiated hESCs indeed occurs through the attachment of cells *via* integrin α5β1, antibody-based cell panning was performed. One hundred milligrams of antibodies specific to the integrin dimers (αIIbβ3 (abcam), αvβ3 (abcam), α4β1 (abcam), and α5β1(Millipore)) were coated in untreated six-well dishes for 1 hr at RT. The excess antibodies were then washed away with 50 mM Tris-HCl, pH 9.5. The plates were finally washed with 5% (v/v) FBS in calcium and magnesium-free PBS to remove residual unattached antibodies and to prevent non-specific attachment of cells. The liquid was removed and cell suspension containing 1×10^5 spontaneous differentiated hESCs was added to the well. The cells were cultured for 12 hr at 37 °C. After 12 hr, the culture dishes were washed with PBS and subjected to DAPI staining and microscopy to visualize individual cells and their attachment to the antibody-coated dishes.

Karyotype Analysis: The karyotypes of the hESCs (H9 cell line at passage 43) and hESC-FN-MSCs (at passage 20) were analyzed based on G-banding with Trypsin using Giemsa (GTG-banding). hESCs and hESC-FN-MSCs were harvested and fixed. The cells at metaphases were karyotyped using a chromosome imaging analyzer system (GenDix, Seoul, South Korea).

In Vitro Detection of Mitochondrial Superoxide Production Using MitoSOX Red and MitoTracker: The ASCs, BMMSCs, and hESC-FN-MSCs at passage 5 and passage 10 were seeded in 12-well dishes containing 12-mm glass coverslips. When the cells reached the desired confluence (typically about 80%), the media were removed from the dishes and prewarmed (37 ^{o}C) Hank's buffered salt solution (HBSS)/Ca/Mg containing 3 μM MitoSOX

Red (Invitrogen) and 500 nM MitoTracker Green (Invitrogen) was added, followed by 15 min incubation at 37 °C. The cells were then washed gently three times with prewarmed (37 °C) HBSS/Ca/Mg. After washing with PBS, coverslips were partially dried and placed on glass slides with mounting medium. Fluorescence images were visualized using a Nikon microscope Eclipse 55i (Nikon, Kanagawa, Japan).

In Vitro Multi-Lineage Differentiation: The ASCs, BMMSCs, and hESC-FN-MSCs at passage 3 were seeded at 1×10^4 cells cm^{-2} on culture plates and cultured in adipogenic, chondrogenic, and osteogenic differentiation media for 2 or 3 weeks. Adipogenic medium was purchased from Gibco BRL (StemPro Adipogenesis Differentiation Kit). The chondrogenic medium was DMEM containing 10% FBS, 1% antibiotics, 1% Insulin-Transferrin-Selenium-A Supplement (Gibco BRL), 50 mM ascorbic acid, 100 nM dexamethasone (Sigma), and 10 ng mL^{-1} transforming growth factor-beta1 (TGFβ1, ProSpec-Tany TechnoGene Ltd., Rehovot, Israel). The osteogenic medium was DMEM containing 10% FBS, 1% glutamax, 1% antibiotics (Gibco BRL), 0.2 mM ascorbic acid, 10 mM glycerol 2-phosphate, and 100 nM dexamethasone (Sigma). The differentiation medium was changed every 2 days.

Histological Analysis: The adipogenic, osteogenic, and chondrogenic differentiation of ASCs, BMMSCs, and hESC-FN-MSCs was evaluated by Oil Red O (Sigma), Alizarin Red S (Sigma), and Alcian Blue (Sigma) staining, respectively. The microscopic images were obtained using microscope (IX71 inverted microscope, Olympus, Tokyo, Japan). For quantitative analysis of differentiation, stained Oil Red O was eluted with isopropanol and quantified by measuring the optical density (O.D.) at 510 nm using a microplate reader (Molecular devices, CA, USA). Alizarin Red S was extracted using 10% (v/v) cetylpyridinium chloride (CPC, Sigma) for the quantification of calcium deposits and the O.D. was measured at 562 nm. Stained Alcian blue was extracted with 6 M guanidine-HCl (Sigma) and quantified by measuring the O.D. at 650 nm.

Teratoma formation: hESCs (1 × 10^6 cells/site, passage 43) and hESC-FN-MSCs (1 × 10^6 cells/site, passage 3) with Matrigel (100 μL, BD Biosciences) were subcutaneously injected into the backs of 8-week-old Balb/c non-obese diabetic/severe combined immunodeficient (NOD/SCID) mice, respectively (*n* = 5 per group). After 10 weeks, teratomas at the injection site were retrieved, dissected, and fixed with 10% formaldehyde in PBS. Paraffin-embedded tissue sections were then prepared and analyzed with Hematoxylin and Eosin (H&E) staining.

References

[1] J. W. Seo, H. Moon, S. Y. Kim, J. Y. Moon, K. H. Jeong, Y. H. Lee, Y. G. Kim, T. W. Lee, C. G. Ihm, C. D. Kim, B. H. Chung, Y. H. Kim, S. H. Lee, PLoS One 2017, 12, e0180045.

Supplementary Figure

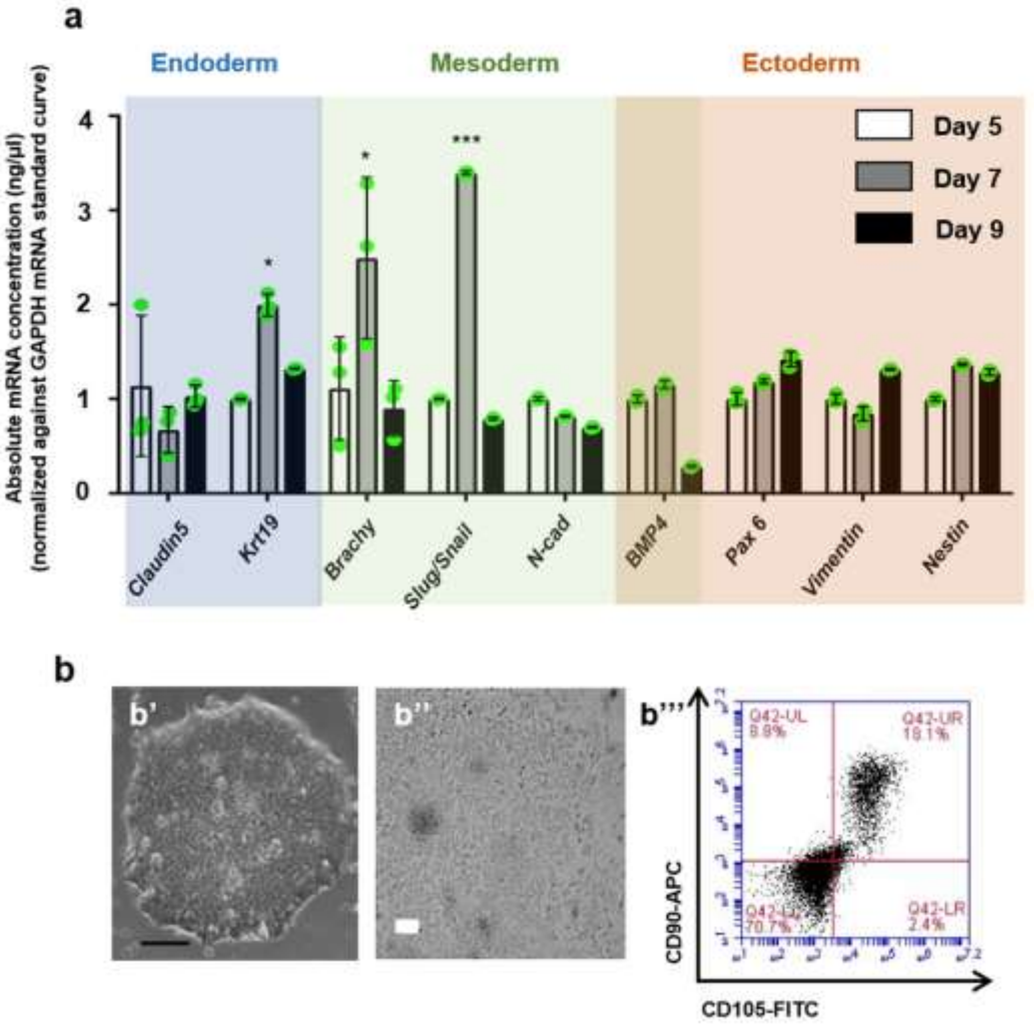

Figure S1. Characterization of spontaneous differentiation from hESCs. a) To optimize the culture period for spontaneous differentiation from hESCs for mesodermal induction, we investigated the changes in three germ layer lineage (endoderm, mesoderm, and ectoderm)-related genes during the progression of spontaneous differentiation from hESCs. Under these spontaneous differentiation conditions, the mesoderm lineage-related markers *Brachy* and *Slug/Snail* were highly expressed after 7 days compared to their expression after 5 and 9 days. $n = 3$, mean ± s.d., $^{*}P < 0.05$, $^{***}P < 0.001$ verse other days (two-way ANOVA). b) Before culturing for spontaneous differentiation, we observed typical undifferentiated hESCs colonies b b'). After 7 days of spontaneous differentiation, we observed successful spontaneously differentiated hESCs with heterogeneous morphology, losing the morphology of a typical undifferentiated hESC colony b b''). Flow cytometry result showed that the proportion of $CD90^{+}CD105^{+}$ cells obtained from spontaneously differentiated hESCs was 18.1% (b b'''). Scale bar: 100 μm.

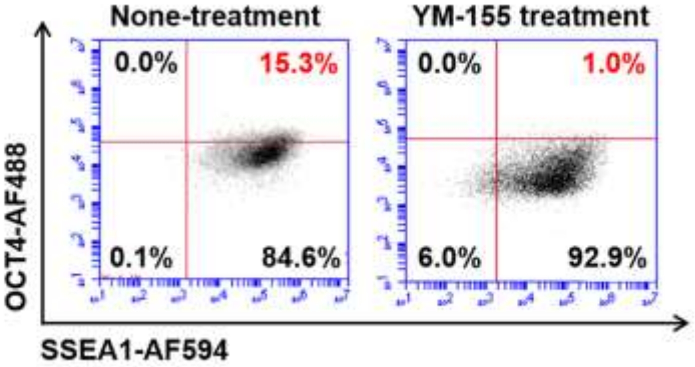

Figure S2. Effect of survivin inhibitor, YM-155, on selective cell death in hESCs. After spontaneous differentiation from hESCs with 10 nM YM-155 treatment for 24 hr, we confirmed a decrease in the pluripotent and undifferentiated hESC marker, OCT4, compared to no treatment.

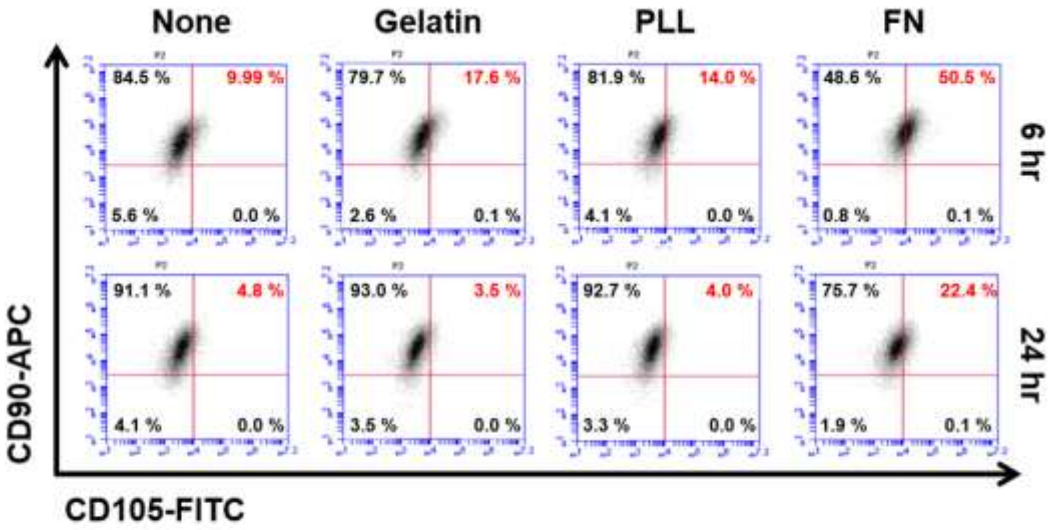

Figure S3. FACS analysis of mesenchymal stem cell markers CD90 and CD105 at 6 and 24 hr after spontaneous differentiated hESC-matrix-mediated binding. FN-coated group shows significantly higher level of positive CD90 and CD105 cells compared to the other groups.

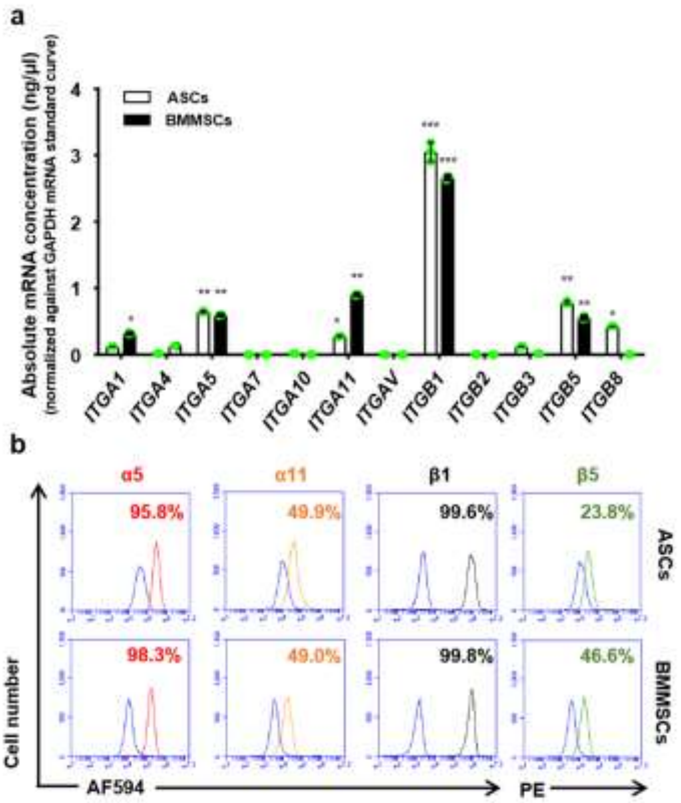

Figure S4. Integrin profile of ASCs and BMMSCs. a) *ITGA5*, *ITGA11*, *ITGB1* and *ITGB5* on ASCs and BMMSCs were dominantly expressed based on the absolute mRNA concentration, determined using normalization against the 18s rRNA mRNA standard curve. n = 3, mean ± s.d., *P < 0.05, **P < 0.01, ***P < 0.001 verse ITGA7 (the lowest expression) on ASCs and BMMSCs (two-way ANOVA). b) The proteins of integrin α5 and integrin β1 on both ASCs and BMMSCs were dominantly expressed, determined with FACS.

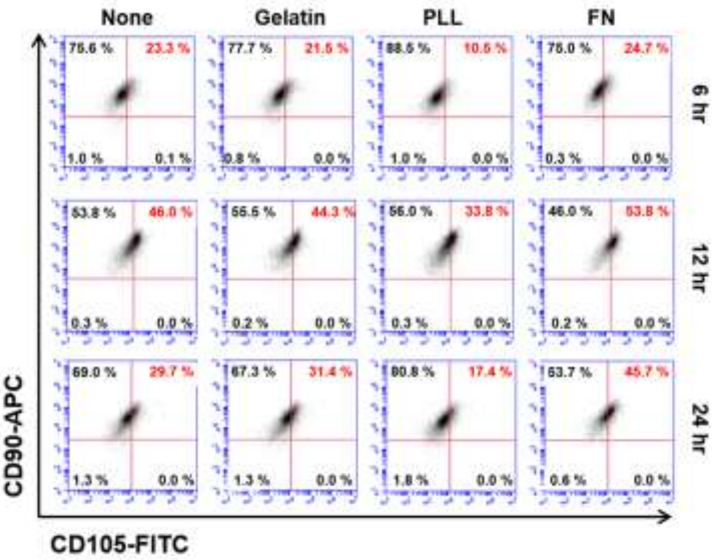

Figure S5. FACS analysis of mesenchymal stem cell markers CD90 and CD105 at 6, 12, and 24 hr after spontaneous differentiated hiPSC-matrix-mediated binding. FN-coated group at 12 hr shows significantly higher level of positive CD90 and CD105 cells compared to the other groups.

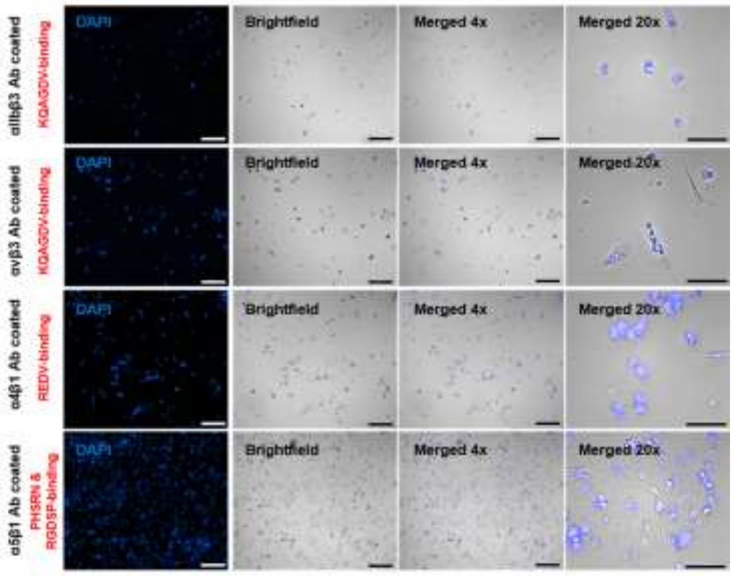

Figure S6. The cell adhesion effect of direct integrin interaction in spontaneously differentiated hESCs using various FN-bound dimer integrin antibody (Ab)-coated plates. We coated various FN-bound dimer integrin Ab, specifically αIIbβ3, αvβ3, α4β1, and α5β1, on culture plates. After 12 hr binding of spontaneous differentiated hESCs to the Ab-coated plates, the integrin α5β1 Ab-coated group showed significantly high cell adhesion compared with the other integrin Ab-coated groups. Scale bar: 100 μm.

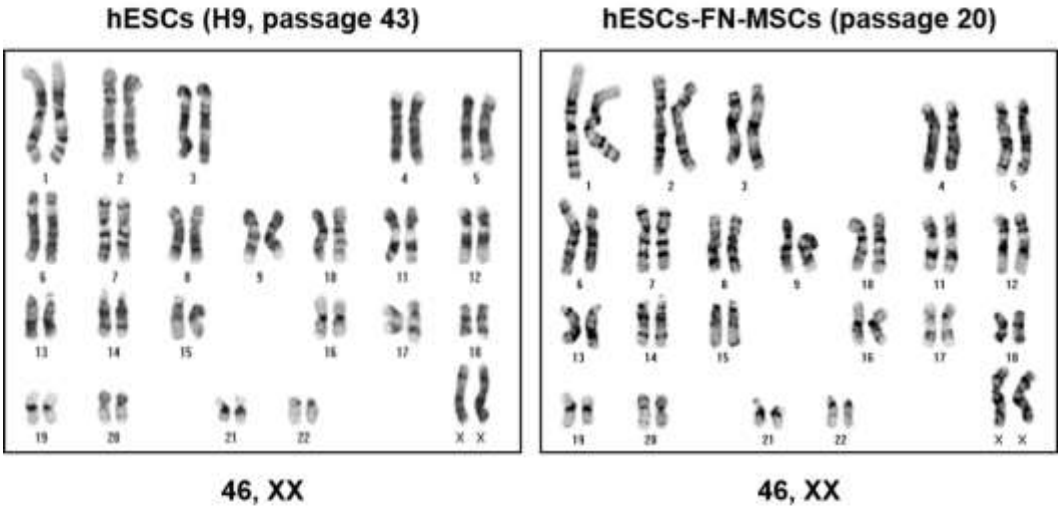

Figure S7. Karyotype profile of hESCs and hESC-FN-MSCs. hESC-FN-MSCs karyotypes were normal when tested at passage 20. All 20 G-banded metaphase cells demonstrated the normal 46, XX karyotype.

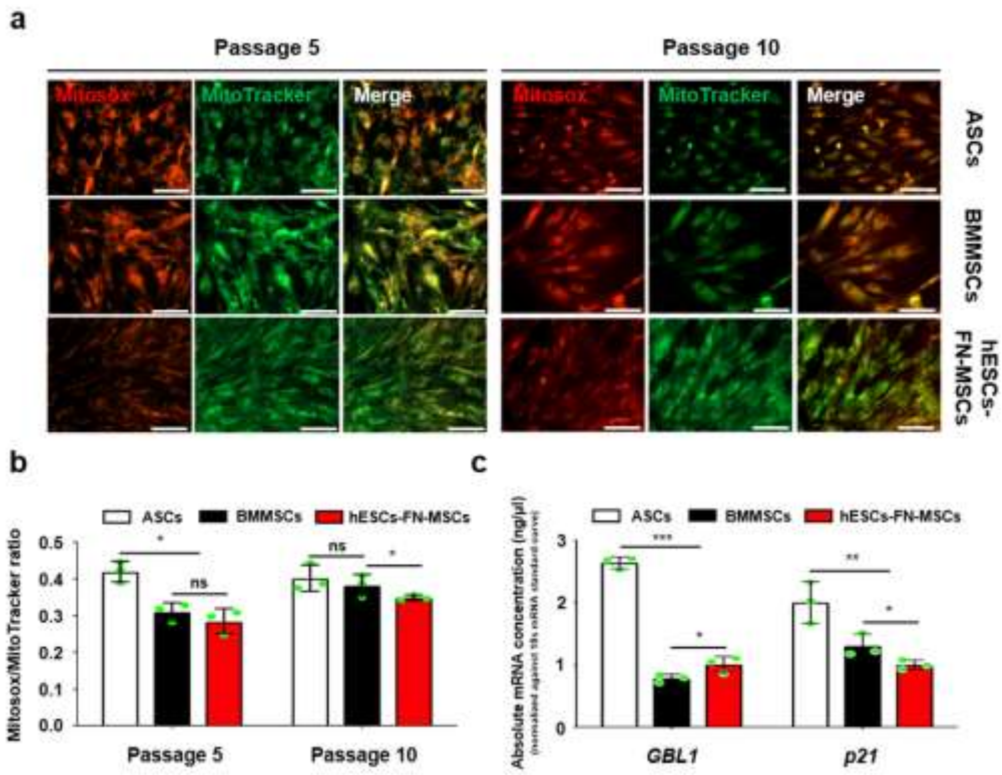

Figure S8. Senescence-associated mitochondrial dysfunction-driven production of reactive oxygen species (ROS). a, b) and senescence associated genes c) such as *GBL1* and *p21* in hESC-FN-MSCs are mostly lower than or similar to those in ASCs and BMMSCs. These results indicated that hESC-FN-MSCs delay the onset of senescence during long-term culture more than ASCs and BMMSCs. Scale bar: 100 µm, mean ± s.d., ns, not significant, *$P < 0.05$, **$P < 0.01$, ***$P < 0.001$ (two-way ANOVA).

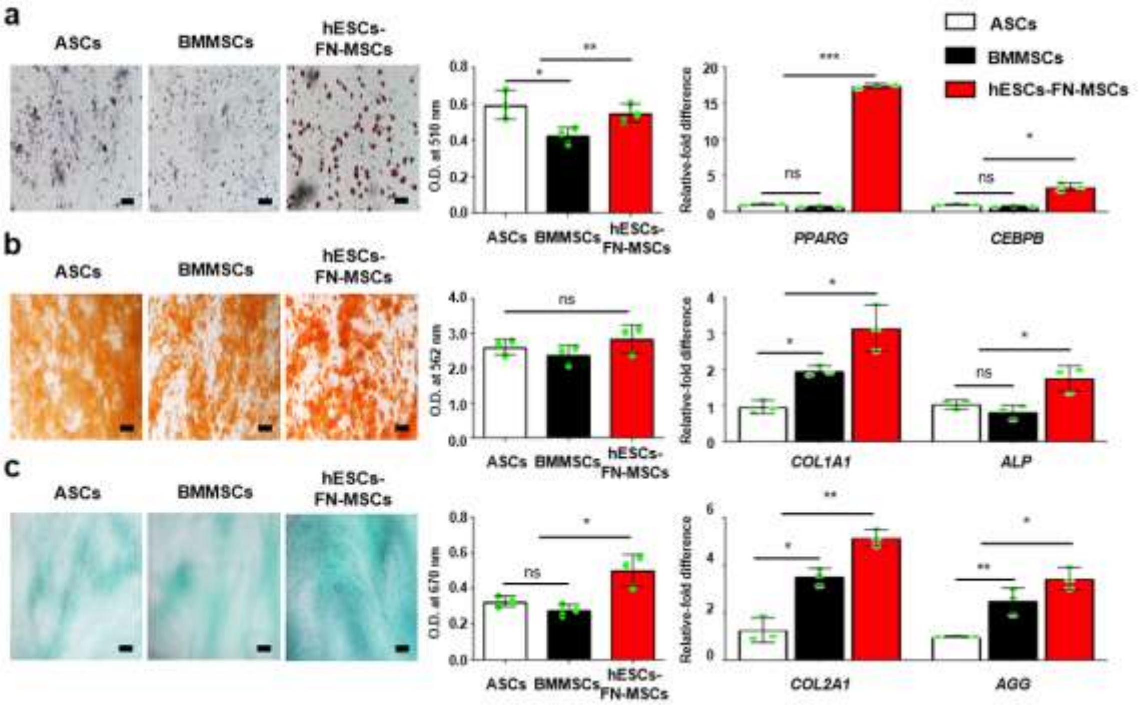

Figure S9. Comparison of *in vitro* multi-lineage differentiation by ASCs, BMMSCs and hESC-FN-MSCs. a) Adipogenic differentiation of ASCs, BMMSCs, and hESC-FN-MSCs evaluated with Oil Red O staining and qRT-PCR (adipogenic marker: *PPARG* and *CEBPB*). Scale bar: 100 µm, $n = 3$, mean ± s.d., ns, not significant, *$P < 0.05$, **$P < 0.01$, ***$P < 0.001$ (one- and two-way ANOVA). b) Osteogenic differentiation of ASCs, BMMSCs and hESC-FN-MSCs evaluated with Alizarin Red staining and qRT-PCR (osteogenic marker: *COL1A1* and *ALP*). Scale bar: 100 µm, $n = 3$, mean ± s.d., ns, not significant, *$P < 0.05$ (one- and two-way ANOVA). c) Chondrogenic differentiation of ASCs, BMMSCs, and hESC-FN-MSCs evaluated with Alcian Blue staining and qRT-PCR (adipogenic marker: *COL2A1* and *AGG*). Scale bar: 100 µm, $n = 3$, mean ± s.d., ns, not significant, *$P < 0.05$, **$P < 0.01$ (one- and two-way ANOVA).

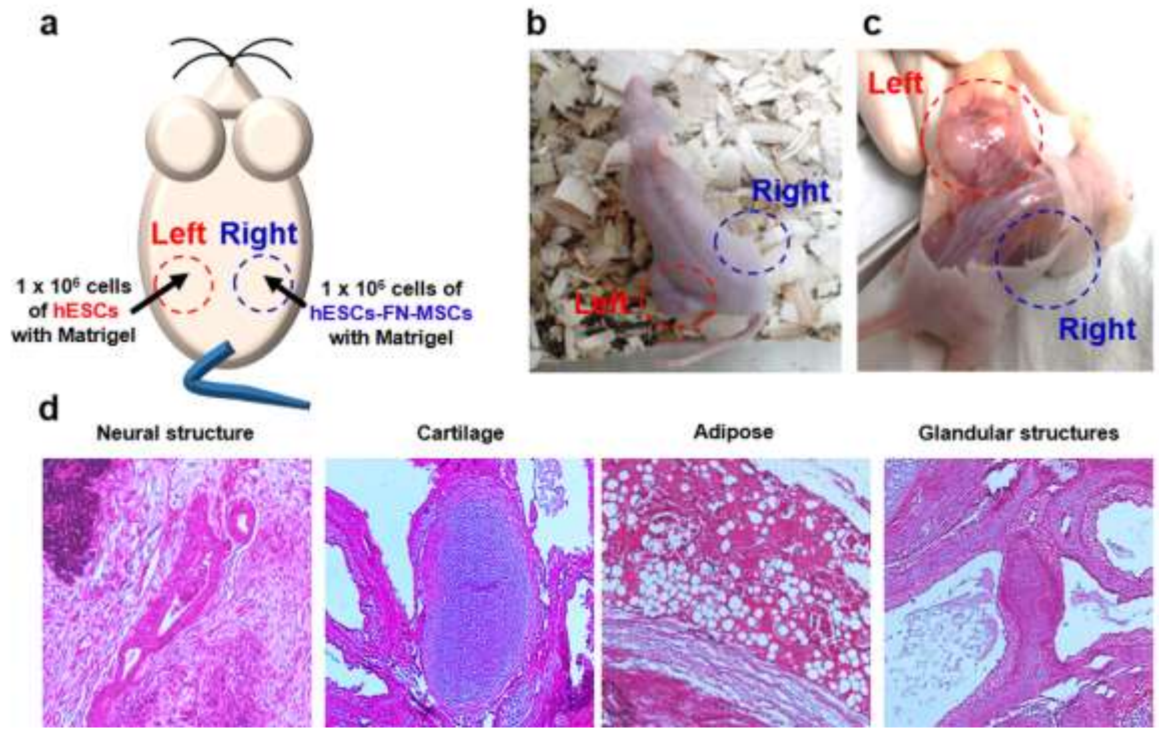

Figure S10. Teratoma formation by hESCs and hESC-FN-MSCs. a) Schematic illustration of implantation of hESCs and hESC-FN-MSCs into the subcutaneous tissue in the back of Balb/c SCID mouse. b, c) Representative image of teratoma formation from hESC- and hESC-FN-MSCs-injected sites. No teratoma formation was observed in Balb/c SCID mouse implanted with hESC-FN-MSCs after 10 weeks. d) hESCs induced teratoma formation including three germ layers. n = 5.

Supplementary Table

Table S1. Primer sequences for qRT-PCR.

**Gene**	**Forward primer (5'-3')**	**Reverse primer (5'-3')**
Claudin5	GTG CTA CAC CCA GTG TGC TG	CCA GTT CAG GTG ACA CCA CTT
Krt19	GCC ACT ACT ACA CGA CCA TCC	CAA ACT TGG TTC GGA AGT CAT
Brachyury	GCA AAA GCT TTC CTT GAT GC	ATG AGG ATT TGC AGG TGG AC
Slug/Snail	TTC CAG CAG CCC TAC GAC CAG	GCC TTT CCC ACT GTC CTC ATC
N-Cadherin	AGC CTG GAA CGC AGT GTA C	GCG AAC CGT CCA GTA GGA T
BMP4	GCA CTA CGG AAT GGC TCC TA	GAG GAA GGA AGA TGC GAG AA
PAX6	GTC CAT CTT TGC TTG GGA AA	TAG CCA GGT TGC GAA GAA CT
Vimentin	CAA GGC GAT GGC CCA GCT GTA A	GTG CGG GTG GAC GTA GTC ACG
Nestin	CCT GGG AAA GGG AGA GTA CC	TGG TCC TTC TCC ACC GTA TC
GLB1	CCT ACA TCT GTG CAG AGT GG	TTC ATC TTG GGC AGA AGG AC
p21	TGG ACC TGT CAC TGT CTT GT	TCC TGT GGG CGG ATT AG
PPARG	GAT ACA CTG TCT GCA AAC ATA TCA CAA	CCA CGG AGC TGA TCC CAA
CEBPB	GCA AGA GCC GCG ACA AG	GGC TCG GGC AGC TGC TT
COL1A1	CCC CTG GAA AGA ATG GAG ATG	TCC AAA CCA CTG AAA CCT CTG
ALP	GAC AAG AAG CCC TTC ACT GC	AGA CTG CGC CTG GTA GTT GT
COL2A1	GGG AGT AAT GCA AGG ACC A	ATC ATC ACC AGG CTT TCC AG
AGG	GCC TGC GCT CCA ATG ACT	ATG GAA CAC GAT GCC TTT CAC
GAPDH	ACA TCG CTC AGA CAC CAT G	TGT AGT TGA GGT CAA TGA AGG G
